# Comparison of free‐energy methods using a tripeptide‐water model system

**DOI:** 10.1002/jcc.25537

**Published:** 2018-10-02

**Authors:** Manuela Maurer, Niels Hansen, Chris Oostenbrink

**Affiliations:** ^1^ Department of Material Sciences and Process Engineering Institute of Molecular Modeling and Simulation, University of Natural Resources and Life Sciences Muthgasse 18, A‐1190, Vienna Austria; ^2^ Department of Energy‐, Process‐ and Bio‐Engineering, University of Stuttgart Institute of Thermodynamics and Thermal Process Engineering Pfaffenwaldring 9, 70569, Stuttgart Germany

**Keywords:** free‐energy calculations, molecular dynamics simulations, active‐site water, GROMOS

## Abstract

We investigate the ability of several free‐energy calculation methods to combine two alchemical changes. We use Bennett acceptance ratio (BAR), thermodynamic integration (TI), extended TI (X‐TI), and enveloping distribution sampling (EDS) to perturb a water molecule, which is restrained to an amino acid that is also being perturbed. In addition to these pairwise methods, we present two two‐dimensional approaches, EDS‐TI and two‐dimensional TI (2D‐TI). We compare feasibility, efficiency and usability of these methods in regard to our simple model system, which mimics the displacement of a water molecule in the active site of a protein on residue mutation. The correct treatment of structural water has been shown to greatly aid binding affinity calculations in some cases that remained elusive otherwise. This is of broad interest in, for example, drug design, and we conclude that thus far, the pairwise method BAR and also the newer X‐TI remain the most suitable methods to treat this problem as long as few end states are involved. © 2018 Wiley Periodicals, Inc.

## Introduction

In recent years, alchemical methods have found widespread use in realistic applications of drug design.^1^, ^2^ Simultaneously, significant progress has been made to predict the presence of water molecules in the active site of proteins, and how these may be displaced on ligand binding.^3^ While rigorous methods are available to predict the binding free energy of individual water molecules,^4^ such predictions are not straightforwardly combined with alchemical methods to compute (relative) binding free energies. Examples of how the appropriate network of waters can improve binding affinity calculations, have been described,^5^ but a systematic analysis of how to most efficiently predict the presence of a water molecule in the context of an alchemical free‐energy calculation seems to be lacking.

The aim of this work is to compare the usability and performance of some common free‐energy methods, using a simple tripeptide as a model system. Alchemical changes to the central side chain of the tripeptide are combined with alchemical changes to a water molecule restrained to this central side chain, creating a system under strain. This setup is intended to identify the most appropriate method to compute relative free‐energy changes which may involve the displacement of an active‐site water molecule, such as when conducting drug design for a specific receptor with water resolved in the crystal structure.

The origins of the model system employed in this work lie in our previous investigations of the oligopeptide‐binding protein A (OppA),^6^ a shuttle protein occurring abundantly in the periplasm of gram‐negative bacteria. OppA is able to bind a broad range of peptide substrates, notably with very low selectivity toward the central amino acid of a series of tripeptides.^7^ OppA's remarkable promiscuity is enabled by a highly ordered network of interfacial water molecules. These serve to mediate interactions between ligand and protein by acting as an adaptable and displaceable buffer to accommodate side chains with various characteristics. The necessary adjustment of this water network in the enclosed active site to side chains of growing sizes was identified as the bottleneck for free‐energy calculations in our previous work.^6^ Here, we evaluate the ability of several methods to predict the presence or absence of an active‐site water molecule during a free‐energy calculation on the ligand.

In the present methodological study, alchemical changes between two of OppA's ligands (tripeptides KGK and KAK) are conducted free in solvent, omitting the protein, which allows for an efficient setup to test a variety of approaches. Rather than explicitly simulating the protein, a distance restraint between a specified water molecule and the site of perturbation in the peptide mimics the spatial confinement of a binding site. We model relative free‐energy changes that may involve displacement of an active site water molecule by performing alchemical changes of the ligand simultaneously with the removal of a water molecule. Here, we test various free‐energy methods and simulation setups to combine both free‐energy changes and investigate calculation efficiency.

Alchemical methods make use of unphysical intermediate states and pathways to calculate the free energies of physical processes. The difference in free energy between two arbitrary states A and B is independent of the specific path connecting them, and of the ability to realize this path or its intermediate states in reality. The efficiency of the calculation may be path‐dependent, however. We compare the Bennett acceptance ratio (BAR) method^8^ to thermodynamic integration (TI)^9^ and its recently proposed extension, extended TI (X‐TI),^10^ which all make use of multiple intermediate states. Furthermore, we will use the enveloping distribution sampling (EDS) method,^11^ in which a single intermediate (or hybrid) state is simulated.

For each of these methods free‐energy differences are calculated between pairs of states, denoting a one‐dimensional change. However, the problem at hand is rather a two‐dimensional problem, involving free‐energy differences due to a change in the peptide and those due to the removal of a water molecule. Rather than explicitly considering only the pairwise free‐energy differences between the four possible end states, we also propose two setups to combine the two dimensions. For two‐dimensional thermodynamic integration (2D‐TI) and the combination method EDS‐TI, free‐energy differences between several states are calculated simultaneously.

Other methods to investigate this two‐dimensional problem exist. A discussion of possible alternatives can be found in the section on Multistate EDS.

## Theory

The alchemical free‐energy methods that make use of intermediate states commonly use a coupling parameter or perturbation coordinate *λ*, to define a path that connects the Hamiltonians of two states A and B. A perturbation is then defined as the gradual change of state A into state B via this coupling parameter, such that sufficient overlap of configurational space between states is ensured. The *λ*‐dependent Hamiltonian *H*(*λ*) is continuous between A and B, and represents state A at *λ* = 0 and state B at *λ* = 1. A typical linear parametrization is:

(1) $$H\left(\lambda ,r,p\right)=\left(1-\lambda \right)H_{A}\left(\lambda ,r,p\right)+\lambda \;H_{B}\left(1-\lambda ,r,p\right)$$

Here, **r** and **p** refer to the positions and momenta of all constituting particles. The Hamiltonians *H*_A_ and *H*_B_ are still functions of *λ* to allow for a soft‐core potential, alleviating the end‐state problem.^12^

### Bennett acceptance ratio

The BAR^8^ method is one approach which makes use of a coupling parameter *λ.* The free energy is estimated from the energy differences between two neighboring states *λ* and *λ* + Δ*λ*:

(2) $$\Delta G_{\lambda ,\lambda +\Delta \lambda }^{\mathrm{BAR}}=k_{B}T\left(\ln\frac{〈f{\left(H\left(\lambda \right)-H\left(\lambda +\Delta \lambda \right)+C\right)〉}_{\lambda +\Delta \lambda }}{〈f{\left(H\left(\lambda +\Delta \lambda \right)-H\left(\lambda \right)-C\right)〉}_{\lambda }}\right)+C$$

To obtain the free‐energy difference, *C* is iteratively calculated until the above ensemble averages, denoted by angular brackets, equal each other. Here,

(3) $$f\left(x\right)=\frac{1}{1+e^{x/k_{B}T}}$$

where *k*_B_ is Boltzmann's constant and *T* is the absolute temperature. For the final free‐energy estimate Δ*G* ^BAR^, the series of free‐energy differences along the intermediate *λ*‐states is summed up. As BAR is often considered to be the optimal free‐energy estimator,^13^ we will use BAR to generate reference free‐energy differences between all relevant states. An additional aim of this work is to identify appropriate alternatives to the BAR method in regard to the posed problem.

### Thermodynamic integration

TI^9^ also makes use of the coupling parameter *λ* to define a path that connects the Hamiltonians *H* of two states A and B. The free‐energy difference along this path is computed from the derivative of the free energy at each single state *λ*, as:

(4) $$\Delta G^{\mathrm{TI}}\left(A\to B\right)=G\left(B\right)-G\left(A\right)=\int_{0}^{1}{〈\frac{\partial H}{\partial \lambda }〉}_{\lambda }\mathrm{d\lambda}$$

The angular brackets denote again an ensemble average, collected from independent equilibrium simulations at varying values of *λ.* This ensemble averaged derivative of *H* with respect to *λ* is numerically integrated for the final free‐energy estimate Δ*G* ^TI^. As the derivative can change strongly with *λ* (a plot that is called a TI profile), the placement of values along *λ* is decisive for the accuracy of Δ*G* ^TI^.

### Extended thermodynamic integration

X‐TI is a recent advancement that alleviates integration errors in TI.^10^ For X‐TI, the derivatives of the Hamiltonian with respect to *λ* at a large number of *λ*_P_‐values, $\frac{\partial H}{\partial \lambda }\lambda_{P}$ are computed on the fly during simulation runs at each simulated point (*λ*_S_), and stored with all other output. To obtain a prediction for any desired nonsimulated value of *λ* (*λ*_P_), these derivatives are reweighed to the appropriate ensemble during an analysis step, according to:

(5) $$〈\frac{\partial H}{\partial \lambda }〉\lambda_{P}=\frac{\left.〈,\frac{\partial H}{\partial \lambda }\right|\lambda_{P}e^{-\left[H\left(\lambda_{P}\right)-H\left(\lambda_{S}\right)\right]/k_{B}T}〉\lambda_{S}}{〈e^{-\left[H\left(\lambda_{P}\right)-H\left(\lambda_{S}\right)\right]/k_{B}T}〉\lambda_{S}}$$

In this way, the entire TI‐profile can be predicted from a few simulated points, the resulting integrand is smooth, and integration errors are minimized on application of eq. (4) to obtain Δ*G* ^X‐TI^.

### Two‐dimensional thermodynamic integration

The concept of thermodynamic Integration can be applied to perturbations of not only one species, but of several. In this work, we conduct the alchemical change of a peptide simultaneously with the alchemical change of a water molecule. A second coupling parameter *κ* is introduced that governs the perturbation of the second species, independent of the first. This equals conducting a series of complete perturbations from *λ* = 0 to *λ* = 1 for the water molecule while keeping the peptide molecule fixed at a specific value of *κ. κ* is then also varied from 0 to 1 in a stepwise fashion, until all combinations of *κ* and *λ* have been visited. This method provides the full free‐energy landscape between the four possible state pairs.

2D‐TI is most easily done by modifying the parametrization of the Hamiltonian as a function of λ. Rather than a linear combination of the end state Hamiltonians *H*_A_ and *H*_B_, as in eq. (1), we formulate the interaction function such that a linear combination of the interaction parameters themselves is used. Using geometric combination rules, the interactions of a (perturbed) atom *i* are described by its partial charge:

(6) $$q_{i}\left(\lambda \right)=\left(1-\lambda \right)q_{i}^{A}+\lambda q_{i}^{B}$$

and the square root of the Lennard‐Jones parameters C12_i_ and C6_i_,

(7) $$\sqrt{C12_{i}\left(\lambda \right)}=\left(1-\lambda \right)\sqrt{\left(C12_{i}^{A}\right)}+\lambda \sqrt{\left(C12_{i}^{B}\right)}$$

(8) $$\sqrt{C6_{i}\left(\lambda \right)}=\left(1-\lambda \right)\sqrt{\left(C6_{i}^{A}\right)}+\lambda \sqrt{\left(C6_{i}^{B}\right)}$$

For a two‐dimensional setup, including soft‐core interactions, we can then define the interaction between two particles i and j as a function of λ and *κ* as.(9)
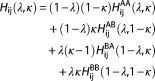
where(10)
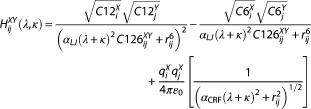
possibly complemented with a reaction‐field contribution. In eq. (10)

(11) $$C126_{\mathrm{ij}}^{\mathrm{XY}}=\frac{\sqrt{C12_{i}^{X}}\sqrt{C12_{j}^{Y}}}{\sqrt{C6_{i}^{X}}\sqrt{C6_{j}^{Y}}}$$

and *α*_LJ_ and *α*_CRF_ are the soft‐core parameters. This formalism reverts back to the parametrization in eq. (1) for interactions between perturbed and nonperturbed particles, but also allows for interactions between two independently perturbed particles.

The derivatives of the Hamiltonian with respect to both *λ* and *κ* are monitored separately in this approach. Thus, the full free‐energy landscape as a function of *κ* and *λ* becomes accessible. For any discrete point on this surface three free‐energy estimates were obtained,

(12) $$G_{1}\left(\lambda +\Delta \lambda ,\kappa +\Delta \kappa \right)=G\left(\lambda ,\kappa \right)+\Delta \lambda 〈\frac{\partial H\left(\lambda ,\kappa \right)}{\partial \lambda }〉\lambda ,\kappa +\Delta \kappa 〈\frac{\partial H\left(\lambda ,\kappa \right)}{\partial \kappa }〉\lambda ,\kappa$$

(13) $$G_{2}\left(\lambda +\Delta \lambda ,\kappa +\Delta \kappa \right)=G\left(\lambda ,\kappa +\Delta \kappa \right)+\Delta \lambda 〈\frac{\partial H\left(\lambda ,\kappa +\Delta \kappa \right)}{\partial \lambda }〉\lambda ,\kappa +\Delta \kappa$$

(14) $$G_{3}\left(\lambda +\Delta \lambda ,\kappa +\Delta \kappa \right)=G\left(\lambda +\Delta \lambda ,\kappa \right)+\Delta \kappa 〈\frac{\partial H\left(\lambda +\Delta \lambda ,\kappa \right)}{\partial \kappa }〉\lambda +\Delta \lambda ,\kappa$$

and the final estimate is obtained by exponential averaging:

(15) $$\Delta G\left(\lambda +\Delta \lambda ,\kappa +\Delta \kappa \right)=-k_{B}T\ln\left[\frac{1}{3}\sum_{i}e^{-G_{i}\left(\lambda +\Delta \lambda ,\kappa +\Delta \kappa \right)/k_{B}T}\right]$$

allowing us to construct the entire landscape starting from G(0,0) = 0. The free‐energy differences Δ*G*^2D ‐ TI^ between the four end states are readily obtained from the differences in free energies at the corners of this landscape.

### Enveloping distribution sampling

EDS^11^ avoids the difficulties and inefficiencies of the above mentioned methods that are associated with having to choose a pathway along a coupling coordinate *λ.* EDS can be considered either an implementation of the umbrella sampling method^14^ as it connects two (or more) phase space distributions through an umbrella biased simulation; or an advancement of the one‐step perturbation method^15^ (which is itself based on Zwanzig's perturbation formula^16^) as it simulates an (optimal) single intermediate reference state. The reference state's Hamiltonian *H*_EDS_ is automatically constructed from the Hamiltonians of the constituent states *i*, however, not as a linear combination, but as an exponential one, that is, the sum of the Boltzmann factors:

(16) $$H_{\mathrm{EDS}}\left(r,p\right)=-\frac{1}{s}k_{B}T\ln\left(\sum_{i=1}^{N_{H}}e^{-s\left(H_{i}\left(r,p\right)-E_{i}^{\mathrm{EDS}}\right)/k_{B}T}\right)$$

where *N*_*H*_ is the number of states or explicit Hamiltonians, *H*_*i*_ *, s* is called the smoothness parameter as it lowers the barrier between states to ease transition, and $E_{i}^{\mathrm{EDS}}$ is the energy offset of each state *i*, used to ensure roughly equal sampling of all states by bringing them all to the same approximate level.^17^ When *i* only consists of two states A and B, only a single energy offset (henceforward simply called *E*) needs to be applied to the higher energy state, and a single smoothness parameter needs to be optimized. Very recently, an alternative functional form to smoothen the EDS energies was proposed which does not make use of an *s* parameter.^18^ This new functional form will not be considered in the current work.

Due to the Boltzmann‐weighted construction of the reference state in eq. (16), the system's Hamiltonian basically follows the Hamiltonians of whichever constituent state currently contributes most to the sum. In other words, the simulated reference state roughly corresponds to either state A or state B at any given time, depending on which of the two results in the lowest energy. Thus, the sampled phase space should be relevant for one of the two states at all times, and the resulting distribution should envelop the distributions of both states A and B. The free‐energy difference Δ*G* ^EDS^ is then computed as.

(17) $$\Delta G^{\mathrm{EDS}}=G_{B}-G_{A}=-k_{B}T\ln\frac{{〈e^{-\left(H_{B}-H_{\mathrm{EDS}}\right)/k_{B}T}〉}_{\mathrm{EDS}}}{{〈e^{-\left(H_{A}-H_{\mathrm{EDS}}\right)/k_{B}T}〉}_{\mathrm{EDS}}}$$

### Enveloping distribution sampling with thermodynamic integration

In EDS‐TI, the change of the peptide is described using TI, while the water is treated using an EDS potential. So instead of starting at a physical end state, the EDS reference state is propagated along the perturbation coordinate *λ.* The derivative of the Hamiltonian with respect to *λ* is obtained from enveloping distributions for the water being present and absent. A simple reweighing of the appropriate properties makes the relevant derivatives at the water end states accessible,

(18) $$〈\frac{\partial H}{\partial \lambda }〉H_{2}O=\frac{{〈{\left.\frac{\partial H}{\partial \lambda }\right|}_{H_{2}O}e^{-\left(H_{H_{2}O}-H_{\mathrm{EDS}}\right)/k_{B}T}〉}_{\mathrm{EDS}}}{{〈e^{-\left(H_{H_{2}O}-E_{\mathrm{EDS}}\right)/k_{B}T}〉}_{\mathrm{EDS}}}$$

and a similar expression for the dummy state. This derivative can then be integrated to obtain the free‐energy differences Δ*G* ^EDS‐TI^ along *λ*, using eq. (4). The free‐energy differences between the two states of water are obtained from eq. (17).

## Methods

### A four‐state model system

**Figure 1:**
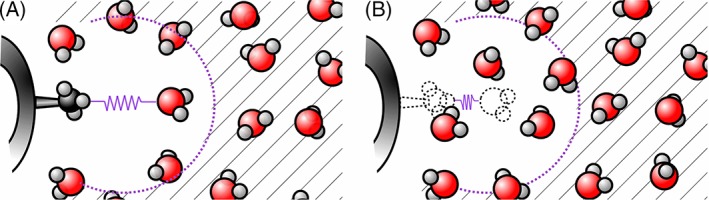
Distance restraint model. a) KAK‐H2O state b) KGK‐Dummy state. The hatched area represents space that could be occupied by protein atoms and is unavailable to the restrained water molecule. [Color figure can be viewed at http://wileyonlinelibrary.com]

In this work, we consider two tripeptides: lysine‐glycine‐lysine, (KGK) and lysine‐alanine‐lysine (KAK). Figure 1 shows a schematic representation of the simulation system. Chemically, the difference between them is a single methyl group, and our calculations, thus, correspond to one of the smallest alchemical changes possible regarding amino acids. The glycine state is created from the alanine state by decoupling all interactions of the single alanine side chain methyl with its surroundings, creating a “ghost” or dummy side chain, while modifying the united‐atom C_α_ atom from a CH to a CH_2_ group. The resulting moiety effectively interacts as KGK. The increased conformational freedom of this state already poses some challenges to the free‐energy methods, as described in our earlier work.^19^

In addition, we consider a water molecule with two different states of existence: “There” or “not there.” The first state is modeled using a regular simple point charge (SPC) water model, the second again by decoupling all its interactions with the surroundings. In either state a distance restraint, which serves as a very simplified model of an active site, pulls that water molecule to the position of alanine's C_β_ (which itself might or might not be interacting with its surroundings). Obviously, a directional distance restraint on a single water molecule is not identical to the pressure in an active site due to crowding with water molecules, but we believe that our simple model captures the most important technical issues regarding the problem of interest quite well. As our analysis is aimed at identifying the most suitable methods and approaches, we only conduct the minimally necessary alchemical changes. In realistic applications more than one water molecule might need to be considered for removal. The approaches employed here could be extended to more elaborate states accordingly. However, our assessment of the different approaches will not be significantly different if one considers one, two or three water molecules. For OppA, the number of water molecules to consider is clearly resolved in the crystal structure; it is a single one in the case of a mutation from Glycine to Alanine.^7^

**Figure 2:**
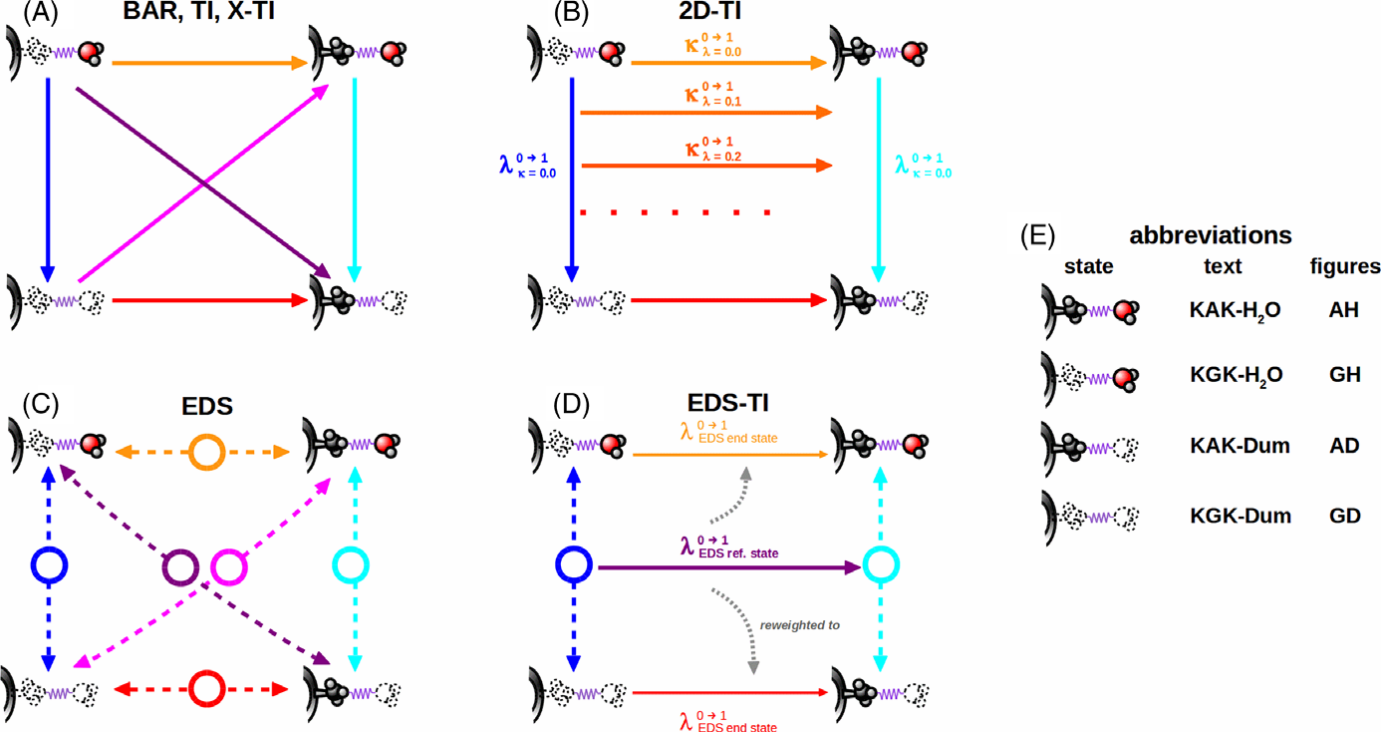
Thermodynamic cycles and schematic method representation. Solid lines represent multistep alchemical methods connecting two states; circles with dashed lines represent one‐step EDS simulations of connected (reference) states. a) Bennett acceptance ratio, thermodynamic integration, and extended thermodynamic integration; b) two‐dimensional thermodynamic integration; c) enveloping distribution sampling; d) enveloping distribution sampling combined with thermodynamic integration. State abbreviations are listed in panel e). [Color figure can be viewed at http://wileyonlinelibrary.com]

Our simple model setup leads to a total of four different overall system states and six possible state pairs, as depicted in Figure 2. Among them, the state pair of KGK‐Dummy ↔ KAK‐Dummy, for example, involves few changes to the system and serves as an easy test case, whereas the state pair of KGK‐Dummy ↔ KAK‐H2O involves the largest changes as well as the introduction of steric clashes, and thus, serves as a difficult test case. Free‐energy differences between all state pairs were calculated using a series of methods (see Theory section and Fig. 2), which in essence showed similar ability to handle the easy test case but differed in their ability to handle the difficult test case.

Figure 2e also lists the system state abbreviations used throughout the text and the condensed form used in figures and tables.

### General simulation settings

All MD simulations were performed using a modified version of the GROMOS11 biomolecular simulation package.^20^ The parameters to describe the involved atomic interactions were taken from the GROMOS 54A8 parameter set.^21^ Covalent bonds were constrained using the SHAKE algorithm,^22^ with a relative tolerance of 10^−4^. During the production simulations, temperature (298 K) and pressure (1 atm) were kept constant by using the weak coupling algorithm,^23^ with coupling times of 0.1 ps and 0.5 ps, respectively. The isothermal compressibility was set to 4.575 x 10^−4^ (kJ mol^−1^ nm^−3^)^−1^.

A time step of 2 fs was used, and at every step short‐range nonbonded interactions up to a distance of 0.8 nm were calculated by means of a pairlist, which was generated every five steps. At every fifth time step intermediate‐range interactions at distances up to 1.4 nm were recalculated and then kept constant between updates. To account for long‐range electrostatic interactions beyond the cut‐off radius of 1.4 nm, a reaction‐field contribution was added to the energies and forces,^24^ using a dielectric permittivity of 61.^25^

The initial starting coordinates for the tripeptide were extracted from the crystal structure of KSK in complex with the OppA protein (PDB code 1B51^7^). The protein was discarded and the tripeptide solvated in a periodic rectangular box containing 2216 SPC water molecules,^26^ with a minimum solute to wall distance of 0.8 nm.

In an initial equilibration phase, the system was heated up to 298 K in three separate simulation steps of 20 ps. In the first two equilibration steps, position restraints were applied on the solute atoms, using force constants of 2.5 x 10^4^ kJ mol^−1^ nm^−2^ and 2.5 x 10^2^ kJ mol^−1^ nm^−2^, respectively.

After an additional 20 ns of equilibration time, we conducted a short perturbation to remove the serine side chain. This dummy side chain was kept throughout to ensure comparability with previous results^6^, ^19^ and allow for future investigations. The closest water molecule adjacent to the C_β_ was chosen to be used for the upcoming perturbations in this work.

We then conducted a preliminary 2D‐TI simulation in which the chosen water was restrained toward the C_β_ position with a harmonic distance restraint using a force constant of 150 kJ mol^−1^ nm^−2^. All other simulations, including a repetition of these preliminary 2D‐TI simulations, were started using the final coordinates of this 2D‐TI test run, and a force constant of 500 kJ mol^−1^ nm^−2^.

Data writeout settings for each method can be found in Table S1 in the Supporting Information.

### Individual settings and remarks for BAR, TI, X‐TI, 2D‐TI, EDS‐TI

A soft‐core potential was used to avoid singularities in forces and *λ*‐derivatives resulting from overlapping atoms during an alchemical transformation.^12^, ^27^ We employed the commonly used softness parameter of *α*_VdW_ = 0.5 and *α*_CRF_ = 0.5 nm^2^ for perturbing the solute water molecule, but *α*_VdW_ = 1.0 and *α*_CRF_ = 1.0 nm^2^ for perturbing the peptide, as described earlier.^6^, ^19^ In EDS‐TI, this softness parameter was used only for the peptide perturbed by TI. While it is more efficient to employ a lower softness due to a reduced curvature of the 〈∂H/∂*λ*〉_*λ*_ curve (necessitating fewer *λ*‐points), we have found previously that an increased softness can be a simple and effective tool to alleviate sampling problems in challenging systems involving end states with unequal conformational flexibility.^19^ Integrated free‐energy differences between the states remain unaffected.^28^, ^29^

As the TI‐profiles of the investigated perturbations were roughly known beforehand,^6^, ^19^ the distribution of simulated points along the perturbation coordinate *λ* could be pre‐optimized accordingly. Each perturbation was run using at least 10 and at most 14 *λ*‐values (see Table S6 in the Supporting Information) spaced manually to cluster in areas of high curvature. For a comparative analysis some of the *λ*‐values were subsequently omitted after the final TI‐profile became known.

After a 100 ps equilibration at one *λ*‐value, a production simulation of 20 ns was started, as well as the equilibration at the next *λ*‐value. For 2D‐TI, the production simulations at any pair of *λ* and *κ* were 1 ns in length, and simulations along *λ* (at a given value of k) were run in parallel.

### Individual settings and remarks for EDS and EDS‐TI

The deciding factor for convergence of free‐energy estimates from EDS simulations is the determination of appropriate parameters for the construction of the reference state. Several search protocols for these parameters exist.^17^, ^30^, ^31^, ^32^, ^33^ As this search must necessarily be conducted prior to production runs, its convergence is essential. Recently, an alternative approach involving replica exchange simulations over multiple values of *s* was suggested.^34^ Because a replica exchange approach invariably involves a significant increase in computational efforts, we do not further explore this option here. Rather, we employed the automated update scheme of Hansen et al.^33^

The *E* parameter was first estimated manually in a short unbiased simulation (we used 200 ps). The result was then used to initialize an automated series of simulations where the system was run for 100 ps during which the *E* and *s* parameters are iteratively updated. The trajectories were analyzed in terms of the energy difference Δ*V*_BA_ between the states A and B: As Δ*V*_BA_ is positive for state A and negative for state B, the sampling per state can be determined. Then, either the *E* or *s* parameter was adjusted accordingly for the next run, with a frequency depending on the current phase of the update scheme. This procedure was iterated at least 200 times (totalling 20 ns or more) for each investigated perturbation, and should lead to optimal parameters, that is, to roughly equal sampling of the constituent states. Production runs of 50 ns length were subsequently conducted with the found parameters.

To construct the EDS reference state describing water in EDS‐TI, the *E* and *s* parameters previously obtained by the automated search protocol were used: At *λ* = 0 (KGK), the parameters for KGK‐H2O ↔ KGK‐Dummy after 200 steps (20 ns) were used. At *λ* = 1 (KAK), the parameters for KAK‐H2O ↔ KAK‐Dummy were used, however, the *E* parameter for KAK‐H2O ↔ KAK‐Dummy was increased by 6 kJ mol^−1^ to reflect the improved search results after 400 steps (40 ns). The *E* and *s* parameters of both perturbations were otherwise virtually identical after 200 and 400 steps, indicating a converged search. At all intermediate *λ*‐points, the *E* and *s* parameters were linearly interpolated. Production runs were 50 ns in length at each *λ*‐point.

The free‐energy change of the EDS reference state along the perturbation coordinate *λ* was obtained by integration of its TI profile according to eq. (4). To calculate the free‐energy change of each EDS end state (H2O or dummy) along the perturbation coordinate *λ* (KGK → KAK), the end state's TI profile must be obtained for integration from the EDS reference state that was propagated along *λ.* To this end, at each *λ*‐point the appropriate free‐energy contribution 〈∂H/∂*λ*〉_*λ*_ was extracted from the reference state and reweighted to the respective end state ensemble (H2O or dummy) according to eq. (18). In each TI end state (KGK or KAK), the free‐energy difference between the EDS end states (H2O or dummy) was obtained from the EDS reference state using eq. (17).

The diagonals in the thermodynamic cycles of Figure 2 were calculated by summing up the respective partial free‐energy changes:

(19) $$\begin{array}{l} \Delta G^{\mathrm{EDS}‐\mathrm{TI}}\left(\mathrm{KGK}-H2O\to \mathrm{KAK}-\mathrm{Dum}\right)=-\Delta G^{\mathrm{EDS}}\left(R_{\mathrm{KGK}}\to \mathrm{KGK}-H2O\right)\\ +\Delta G^{\mathrm{TI}}\left(R_{\mathrm{KGK}}\to R_{\mathrm{KAK}}\right)+\Delta G^{\mathrm{EDS}}\left(R_{\mathrm{KAK}}\to \mathrm{KAK}-\mathrm{Dum}\right) \end{array}$$

and

(20) $$\begin{array}{l} \Delta G^{\mathrm{EDS}‐\mathrm{TI}}\left(\mathrm{KGK}-\mathrm{Dum}\to \mathrm{KAK}-H2O\right)=-\Delta G^{\mathrm{EDS}}\left(R_{\mathrm{KGK}}\to \mathrm{KGK}-\mathrm{Dum}\right)\\ +\Delta G^{\mathrm{TI}}\left(R_{\mathrm{KGK}}\to R_{\mathrm{KAK}}\right)+\Delta G^{\mathrm{EDS}}\left(R_{\mathrm{KAK}}\to \mathrm{KAK}-H2O\right) \end{array}$$

where *R*_KGK_ and *R*_KAK_ represent the EDS state at *λ* = 0 and *λ* = 1 respectively.

### General data analysis

Error estimates were obtained from block averaging,^35^ except for X‐TI, which (for technical reasons) used the bootstrapping method (employing 100 bootstraps). Error estimates on sums of free‐energy differences and from numerical integration were obtained using error propagation.

For the coupling‐parameter‐based methods, the six perturbations in this work were run using 12 to 14 *λ*‐points (see Table S6 in the Supporting Information). In a first analysis step, to reduce convergence time estimates and provide comparability, 10 *λ*‐points were manually selected for each perturbation such that the resulting free‐energy estimate was within ½ *k*_B_ *T* of the (12 or) 14 *λ*‐point estimate. This was verified for BAR, TI, X‐TI, and EDS‐TI at simulation lengths of 1 and 20 ns per *λ*‐point (data not shown). All further analyses conducted here were performed using the data of these 10 *λ*‐points only, unless explicitly mentioned otherwise.

To assess the quality of the free‐energy calculations we examined the cycle closure. As free energy is a state function, the sum of a series of perturbations leading back to the original state must always amount to zero. In Figure 2, four different three‐membered cycles and one four‐membered cycle can be constructed for the system at hand. All individual cycle closure values were determined for the maximum available simulation time as well as for reduced simulation sets.

There are *N*_cycles_ thermodynamic cycles *O*, each built up from $N_{\text{legs}}^{O}$ serially conducted perturbations *j.* For better comparability we compute Σ*,* the sum of all cycle closure deviations from 0 over all thermodynamic cycles *O*, and define the average deviation per perturbation Ω as:

(21) $$\Omega =\frac{1}{N_{\text{cycles}}}\sum_{O}^{N_{\text{cycles}}}\frac{1}{N_{\text{legs}}^{O}}\left|\sum_{j}^{N_{\text{legs}}^{O}}\Delta G_{j}\right|$$

Ω can be expected to become smaller with increasing amounts of sampling.

Additional quality assessments that are specific to each method will be listed in the Results and Discussion section.

### Retrospective analysis: Convergence

To assess the shortest necessary simulation time for each method to yield satisfying results, first a free‐energy target value (accuracy) plus allowed uncertainty (precision) were chosen. Roughly following the work of Bruckner and Boresch^36^ we investigate the minimal simulation time needed to obtain an estimate that is within ½ *k*_B_ *T* of a reference value, and an uncertainty smaller than ½ *k*_B_ *T.*

As BAR is often seen as the best estimator for free‐energy calculations,^13^ the result from the longest available simulation for each perturbation employing BAR, using all available *λ*‐points, was taken as the target value to be achieved by all methods. This provides a common frame of reference for all methods, but for some methods the results are systematically or at least initially too far apart to reach convergence within the available simulation time, despite yielding stable free‐energy estimates and low error estimates. In these cases, we have additionally studied the self‐convergence and used the method's own result calculated from its longest available simulation as the target value. This provides a measure of how long a method needs to produce consistent estimates.

For each method, a first estimate was calculated after minimum simulation time (1 ns, or 1 ns per *λ*‐point) and then compared: If this estimate deviated from the reference value by more than ½ *k*_B_ *T*, or if the associated error estimate was larger than ½ *k*_B_ *T*, the simulations were “prolonged,” meaning that another ns of the already existing trajectory was added and a new cumulative estimate calculated. This was repeated until the resulting value either fell within the defined boundaries, or the maximum simulation length had been reached.

The process of adding more data (i.e., simulation time) to the free‐energy estimate is straightforward for EDS where only a single trajectory exists. For the coupling parameter‐based methods, however, separate simulation trajectories exist for each discrete coupling parameter value; thus, a choice has to be made as to which *λ*‐points to prolong.

To this end, we divided the maximum allowed overall deviation and overall error evenly between the number of contributions to the free‐energy estimate to get the maximum allowed deviation and error per contribution. (This represents a slightly stricter error criterion than distributing the squared error.) For TI, and X‐TI, there is one contribution per *λ*‐point (i.e., N_*λ*_ = 10). For BAR, there is one contribution per interval between *λ*‐points (*N*_*λ*_‐1). The allowed maximum deviation per contribution was then compared with the current contribution to the free‐energy estimate at each prolongation step, resulting in the following selection criteria:

*1. Accuracy criterion:* The current contribution differed from its final value by more than the allowed deviation per contribution.

(22) 〈∂H/∂λ〉λ,t−〈∂H/∂λ〉λ,20nsΔλi>12kBTNλforTIandX‐TI

(23) ΔGλ,λ+Δλ,tBAR−ΔGλ,λ+Δλ,20nsBAR>12kBTNλ−1forBAR

*2. Precision criterion:* The current contribution had an associated effective error larger than the allowed error per contribution.

(24) σ〈∂H/∂λ〉λ,tΔλi>12kBTNλforTIandX‐TI

(25) σΔGλ,λ+Δλ,tBAR>12kBTNλ−1forBAR

Here, Δλi=12λi−λi−1+λi+1−λi, as for TI and X‐TI, each *λ*‐point governs an interval on the TI profile of half the distance to the points flanking it. Each *λ*‐point's calculated free‐energy derivative and associated error are, thus, weighted (multiplied) by this distance to give its effective contribution to the integral.

First, if a contribution according to criterion 1 was identified, the corresponding simulation was prolonged. Both *λ*‐points flanking an interval were prolonged for BAR. If more than one point (or pair of points for BAR) met the criteria, only the largest violation was registered at each step of the prolongation algorithm, and the process repeated until no more points fulfilling criterion 1 could be identified. Then, the process was repeated by identifying *λ*‐points according to criterion 2. Once the overall convergence criteria were met, the trajectory lengths of all *λ*‐points was totalled.

### Predictive analysis, and prolongation algorithm for BAR, TI, X‐TI, EDS‐TI

For all methods investigated, a predetermined amount of simulation time was initially used (20 ns per *λ*‐point, or 50 ns for EDS). This is especially common for the coupling‐parameter‐based methods, where available simulation time simply gets evenly distributed among all *λ*‐points. However, it would be more efficient to spend computation time only on slowly converging contributions. The problem is that identifying such contributions according to the accuracy criterion (criterion 1) is usually not possible when starting a regular free‐energy calculation, since reference values are unavailable.

Identifying contributions only according to a variant of the precision criterion (criterion 2) is one possible approach when deciding on the fly which simulations to prolong. A simple guideline to “predict” where to efficiently invest additional computational resources is to select the simulation—or pair of simulations in the case of BAR—yielding the largest effective error (i.e., $\sigma_{{〈\partial H/\partial \lambda 〉}_{\lambda ,t}}\Delta \lambda_{i}$ for TI and X‐TI). Given a fixed overall “computational budget” of 20 ns, here, we conduct such a predictive analysis to further study convergence.

This error criterion can only be applied to already (manually) established values of *λ.* Automated methods that are also able to suggest the placement of additional *λ*‐points interspersed between already simulated ones to optimally lower the resulting integrated error are currently being developed by Stroet and Mark.^37^ They are, however, beyond the scope of the present work.

## Results and Discussion

### Preparatory simulations

The coupling‐parameter based approaches are relatively straightforward to use. Before running the production simulations, the user has to decide on an initial set of *λ*‐values to simulate at, and to select an appropriate equilibration scheme. After simulating this initial set, additional *λ*‐values may be added, or simulations prolonged, to achieve converged results.

For EDS, a more elaborate initial parameter search is required to construct a reference state Hamiltonian that samples each of the constituent end states roughly equally. To construct an EDS reference state Hamiltonian that spends equal sampling time in each of the constituent end states, two parameters have to be optimized beforehand: The energy offset between the states *E*, and the factor lowering or smoothing the energy barrier between the states *s* need to be determined empirically. These two parameters are not independent of each other, which makes a carefully alternating adjustment protocol necessary to find a suitable set. Although several schemes have been developed,^17^, ^30^, ^31^, ^32^, ^33^ this parameter search is currently and necessarily the bottleneck of the method, and the simulation time spent on it must be added to any simulation time spent on the actual production runs for a proper estimate of the minimum necessary convergence time.

The exact value of *E* has somewhat less impact on sampling efficiency than the exact value of *s.* For example, the free‐energy difference associated with the transformation of a methane into a water molecule is rather insensitive to a change in the *E* parameter over a range of 15 < *E* < 40 kJ mol^−1^, that is, much larger than the remaining fluctuations at the end of the parameter update simulations (data not shown). Thus, in the present work we assessed parameter search convergence mainly based on the *s* parameter. As we expected the possibility of requiring a rather low *s* parameter, we started the search procedure from the unusually low value of *s* = 0.002.

The employed automated search scheme^33^ adjusts and updates the *E* and *s* parameters necessary for successful sampling with the EDS method by a certain percentage value in a series of iterative steps. For all perturbations the search scheme was initially run for 200 steps (à 100 ps), and the resulting parameter values, converged or not, used for the production runs to stay within a reasonable time scale.

A first production run using *E* and *s* parameters obtained after 200 steps (20 ns) of the search scheme yielded clearly flawed results for the KGK‐Dummy ↔ KAK‐H2O perturbation (data not shown), so the search was prolonged and a second production run using the *E* and *s* parameters obtained after ~400 steps of the search scheme was conducted. An additional manual search including the individual analysis of sampled distributions and manual parameter adjustment after each step^33^ did also not yield more suitable parameters (data not shown). All data in this work concerning the KGK‐Dummy ↔ KAK‐H2O perturbation calculated with EDS pertain to the second production run after 40 ns parameter search.

**Figure 3:**
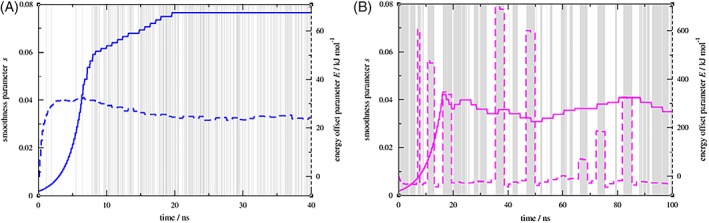
EDS parameter evolution during the automated search. a) For KGK‐H2O ↔ KGK‐Dummy, convergence was reached just before 20 ns simulation time. b) For KGK‐Dummy ↔ KAK‐H2O, convergence was not reached within 100 ns. White background areas signify that the reference state currently samples state A, gray areas signify state B. Both panels show a total of about 12,000 transitions. Note that, especially for panel a, most of the transitions have a lifetime below the resolution of this figure, and that, especially for panel b, many of the sampled configurations are not actually relevant to the end state they are attributed to. [Color figure can be viewed at http://wileyonlinelibrary.com]

To verify whether the used *E* and *s* parameter values had indeed converged, all search procedures were then prolonged, up to a maximum of 1000 steps. Two characteristic parameter evolution plots are presented in Figure 3. The parameter evolutions for all six perturbations at maximum search scheme length can be found in Figure S1 in the Supporting Information, and an overview over all values in Table S2 in the Supporting Information.

For KGK‐Dummy ↔ KAK‐H2O, the set of *E* and *s* parameters was still not converged after 100 ns automated search procedure (100 ns simulation time), as can be seen in Figure 3. After each transition to the KAK‐H2O state, the scheme attempts to offset the highly unfavorable interaction caused by overlap of atomic radii with a huge *E* parameter. In addition, to ease transitions between the two very different states, the smoothness parameter must be chosen so low that the resulting reference state Hamiltonian does not sample the relevant parts of phase space anymore (see also the discussion for the production runs below). Judging from Figure 3 it is likely that any amount of further parameter search simulations will not lead to more appropriate parameters for this case, without first modifying the iterative update procedure itself.

For KGK‐Dummy ↔ KAK‐Dummy the final *s* parameter value yielded by the search procedure is extraordinarily high (~0.8, see Table S2 and Fig. S1 in the Supporting Information), meaning that this perturbation is especially easy to handle for the EDS method. Due to this extraordinarily high *s* parameter value, the search scheme simply takes some time to reach this plateau from our low starting point of 0.002. It might not even have reached its converged value within the prolonged search time of 100 ns. Although the *s* parameter value that was used for the production run (~0.5) was, thus, not yet converged after the 20 ns search scheme, it is still by far the highest value of the set.

### Results at maximum simulation length

The simple model system employed here is intended to model the replacement of a water molecule in a highly confined binding site, approximated by an artificial distance restraint. The aim of our analysis is to identify the most appropriate alchemical free‐energy method for tackling the problem at hand.

Free‐energy difference estimates for all six perturbations calculated with all methods including all available data are given in Table 1. Almost all methods agree within ~1 kJ mol^−1^ with each other, and only EDS deviates by more than 1 *k*_B_ *T* from the other methods for the difficult test case, KGK‐Dummy ↔ KAK‐H2O (GD‐AH).

**Table 1:** Free‐energy differences (kJ mol^‐1^) at maximum simulation length for the individual perturbations.

	GH ‐ AH	GD ‐ AD	GH ‐ GD	AH ‐ AD	GH ‐ AD	GD ‐ AH
*Pairwise methods*						
BAR	17.0 ± 0.4	3.6 *±* 0.2	16.9 *±* 0.04	3.3 *±* 0.2	20.8 *±* 0.1	0.0 *±* 0.2
TI	16.9 ± 0.4	3.6 *±* 0.3	16.5 *±* 0.3	2.3 *±* 0.3	20.3 *±* 0.4	1.2 *±* 0.4
X‐TI	17.0 ± 0.03	3.5 *±* 0.02	16.9 *±* 0.04	3.4 *±* 0.05	20.8 *±* 0.05	−0.1 *±* 0.1
EDS	16.9 ± 0.2	3.9 *±* 0.2	16.6 *±* 0.1	1.3 ± 0.6	21.9 ± 0.4	2.7 ± 0.7
*Multistate methods*						
EDS‐TI	18.1 ± 0.04	4.3 *±* 0.1	16.7 ± 0.1	1.0 *±* 0.5	19.8 ± 0.3	2.1 ± 0.5
2D‐TI	16.8 ± 0.8	3.2 *±* 0.6	17.0 *±* 0.7	3.0 *±* 0.7	19.8 *±* 1.0	−0.2 *±* 1.8

Coupling parameter methods use all available *λ*‐points.

An overview over all total production run times is given in Table S3 in the Supporting Information. Additional simulation time that had to be spent on preparatory steps is further discussed in the section on the Retrospective analysis:

In Table 2, cycle closure is calculated. (Production run times for these cycles are given in Table S4 in the Supporting Information.) For BAR, TI, and X‐TI, each of the five cycles closes with < ½ *k*_B_ *T* deviation from 0 at maximum simulation length. For BAR and X‐TI, each of the five even closes within 0.5 kJ mol^−1^. For EDS, the worst cycle only closes within 3.7 kJ mol^−1^. The values for Σ and Ω are largest for EDS and EDS‐TI, suggesting large inconsistencies within the various simulations.

**Table 2:** Free‐energy differences (kJ mol^‐1^) at maximum simulation length along the thermodynamic cycles.

	4‐circle	GH‐GD‐AH	GH‐GD‐AD	AH‐AD‐GD	AH‐AD‐GH	Σ	Ω
*Pairwise methods*							
BAR	−0.2 *±* 0.5	−0.1 *±* 0.5	−0.3 *±* 0.2	−0.3 *±* 0.3	−0.5 *±* 0.5	1.4 ± 0.9	0.1 ± 0.2
TI	−0.8 ± 0.6	0.7 ± 0.6	−0.2 ± 0.6	−0.1 *±* 0.6	−1.1 *±* 0.6	3.0 ± 1.3	0.2 ± 0.3
X‐TI	−0.1 *±* 0.1	−0.2 *±* 0.1	−0.4 ± 0.1	−0.3 *±* 0.1	−0.5 *±* 0.1	1.5 ± 0.2	0.1 ± 0.05
EDS	−2.3 *±* 0.7	2.4 *±* 0.7	−1.4 *±* 0.4	0.1 *±* 0.9	−3.7 *±* 0.8	10.0 ± 1.6	0.6 ± 0.4
*Multistate methods*							
EDS‐TI	−1.9 *±* 0.5	0.7 *±* 0.5	1.2 *±* 0.3	−1.2 *±* 0.7	−0.7 *±* 0.6	5.8 ± 1.2	0.4 ± 0.3
2D‐TI	−0.4 *±* 1.4	0.0 *±* 2.1	0.4 *±* 1.4	−0.4 *±* 2.0	0.0 *±* 1.5	1.2 ± 3.8	0.1 ± 1.0

Simulation data is taken from Table 1. Coupling parameter methods use all available *λ*‐points.

Production run time for EDS, however, is much shorter than for the other methods, even when including the preparatory parameter search time. A comparison of cycle closure, Ω and Σ at equal total simulation lengths (production run time and preparatory parameter search time) will be discussed below.

### Additional quality assessments at maximum simulation length

As an additional quality assessment for TI, the last snapshot after equilibration has been taken as input to reverse the alchemical change just conducted, that is, the perturbations were also run in full length for all *λ*‐points in backward direction. This backward simulation represents a minimal thermodynamic cycle, and any substantial deviations of it evaluating to 0 can be signs of a shift in the sampled ensemble. The difference of equivalent points in forward and backward directions (hysteresis) is given in Table S5 and exemplified in Figure S5 in the Supporting Information by plotting the backward TI‐profile with inverted values (*y* → ‐*y*).

In all six perturbations calculated with TI, the deviation between forward and backward free‐energy estimates was below 0.4 kJ mol^−1^ at maximum simulation length. The diagonals of the thermodynamic cycle, KGK‐H2O ↔ KAK‐Dummy and KGK‐Dummy ↔ KAK‐H2O, showed the largest hystereses of the set, indicating unsurprisingly that those were the most difficult perturbations to handle for TI. Even at minimum simulation length (1 ns per *λ*‐point, 10 *λ*‐points) the hysteresis was below ½ *k*_B_ *T* for all transitions except for the KGK‐H2O ↔ KAK‐Dummy perturbation, where it amounted to 1.9 kJ mol^−1^.

**Figure 4:**
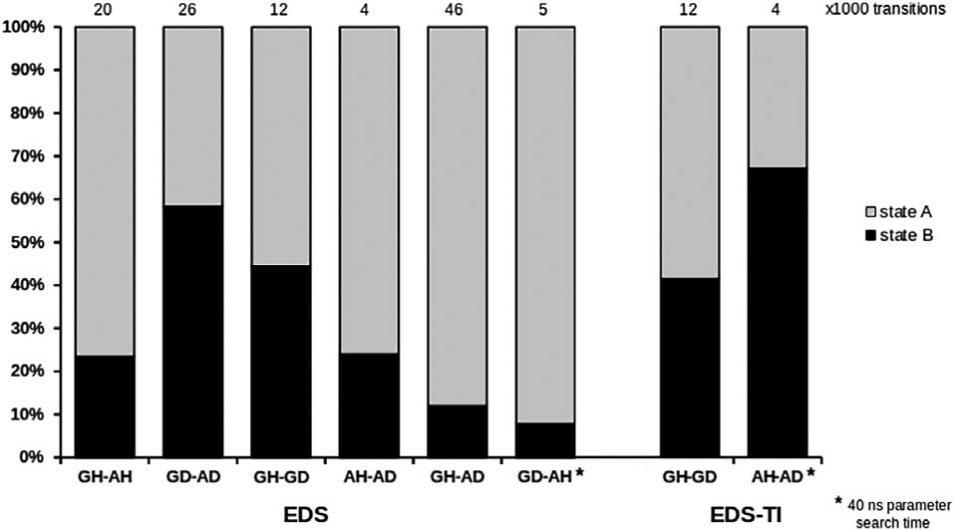
Sampling ratio for EDS production runs and the EDS part of EDS‐TI during 50 ns. The number above the bars refers to the number of transitions between the two states, given in units of 1000. All use the parameters after 20 ns search time, except the GD‐AH case for EDS and the AH‐AD case for EDS‐TI, which used updated parameters after 40 ns search time.

As an additional quality assessment for EDS and EDS‐TI, Figure 4 shows the sampling ratio^32^ and the number of state transitions in the production runs. Only the two easiest perturbations, KGK‐Dummy ↔ KAK‐Dummy (GD‐AD) and KGK‐H2O ↔ KGK‐Dummy (GH‐GD) come close to the ideal EDS sampling ratio of 50% simulation time per state. As was described above, the *s* parameter used for the KGK‐Dummy ↔ KAK‐Dummy case was probably still improvable, although obviously easily sufficient for appropriate sampling.

In EDS‐TI, water is treated by constructing an EDS reference state that is propagated along *λ* by TI. An easy case, the KGK‐H2O ↔ KGK‐Dummy (GH‐GD) perturbation, is used as the *λ* = 0 starting state. Its balanced sampling ratio and adequate number of state transitions are virtually identical to the pure EDS case, since it is essentially just a repetition of that simulation. The KAK‐H2O ↔ KAK‐Dummy (AH‐AD) perturbation at the TI end state *λ* = 1 is much harder to handle for EDS. Its sampling ratio is again suboptimal in EDS‐TI, but closer to the ideal ratio by about 10 percentage points than in EDS. This is caused by the improved energy offset parameter *E* (the parameter search time was doubled for EDS‐TI; the value of *s* stayed virtually identical). This improvement also causes the preferred water state to switch from H2O to dummy.

Unsurprisingly, the difficult KGK‐Dummy ↔ KAK‐H2O (GD‐AH) case shows the least balanced sampling and almost the lowest number of state transitions, even after spending additional time on the parameter search. Spending only about 10% of the simulation time in the KAK‐H2O state is, with ~5000 transitions to the KGK‐Dummy state, still sufficient to compute a free‐energy difference within 2.7 kJ mol^−1^ of the BAR reference value. Prolongation of the simulation is not expected to lead to a closer match here; the discrepancy between the BAR reference value and the EDS estimate is more likely the result of insufficient overlap between the EDS reference state and the KAK‐H2O state. This means that many sampled configurations attributed to an end state after a transition are not highly relevant to that end state.

**Figure 5:**
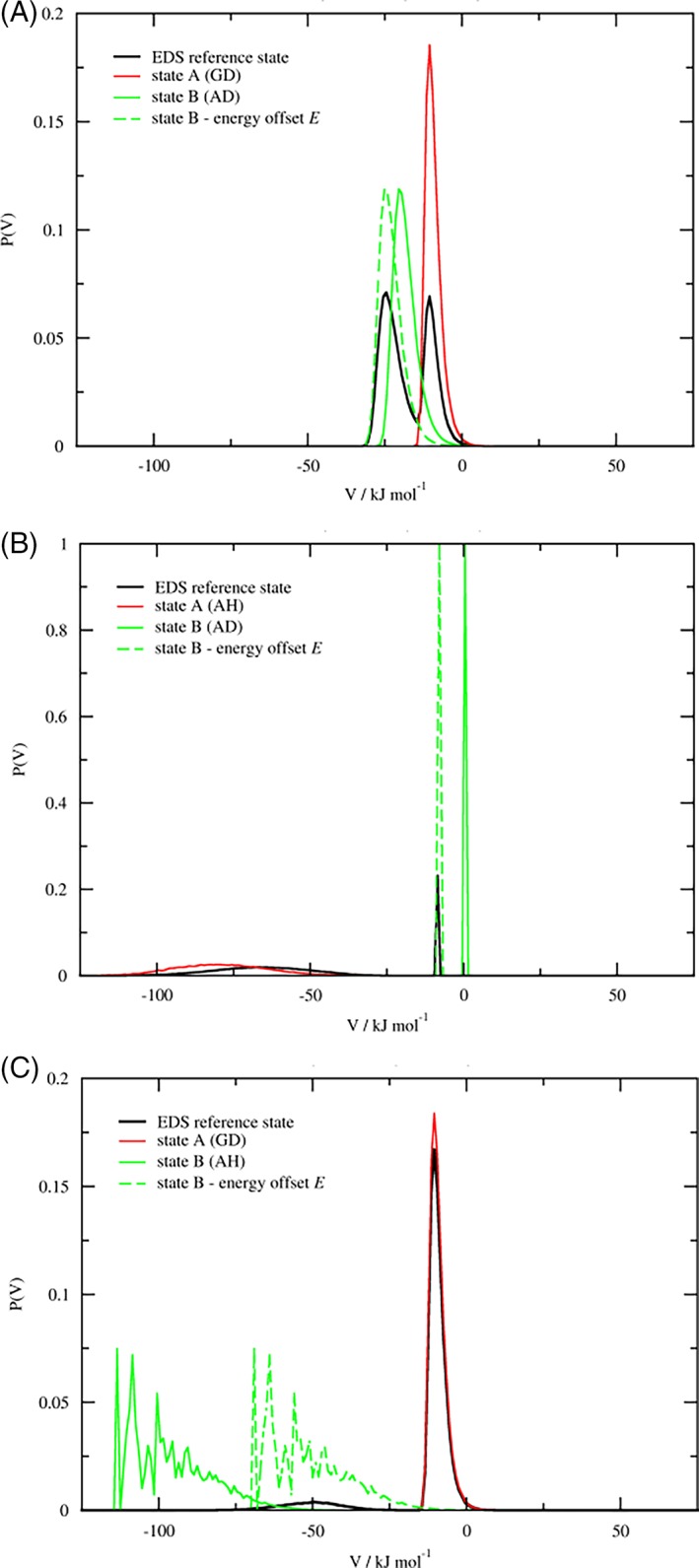
EDS energy distributions. Potential energy of the perturbed atoms for a) KGK‐Dummy ↔ KAK‐Dummy (GD‐AD), b) KAK‐H2O ↔ KAK‐Dummy (AH‐AD), c) KGK‐Dummy ↔ KAK‐H2O (GD‐AH), during 50 ns production run. The distribution of the EDS reference state (black) is compared to the reweighted energy distributions of the end states (red and green). Note that the dummy state (green) in panel b has its width given by the distribution bin width only.

This is demonstrated in Figure 5, where the EDS energy distributions of selected transitions are compared to the reweighted energy distributions of the end states, possibly shifted by the energy offset. Corresponding figures for all remaining perturbations can be found in Figure S4 in the Supporting Information. In panels 5a and 5b it is shown that the EDS reference state samples the real‐state energy distribution fairly well. However, in panel 5c that shows the KGK‐Dummy ↔ KAK‐H2O perturbation, the latter state is only sampled infrequently and the energy distribution of the end state is rather noisy and ill‐defined.

### Retrospective analysis: Minimum simulation length required for convergence

In Table 3, the total amount of simulation time necessary to fulfill the convergence criteria and algorithm outlined in the Methods section is given. 2D‐TI cannot be expected to perform competitively due to the large number of intermediate states that are sampled; using a minimum of 1 ns per simulation with 10 x 10 *λ*‐points, this approach still requires at least 100 ns.

**Table 3:** Minimum simulation time (ns) required to converge to the BAR reference data (or own end value, in the last rows).

	GH‐AH		GD‐AD		GH‐GD		AH‐AD		GH‐AD		GD‐AH		All 6	
*Pairwise methods*	t_sim	+ pre	t_sim	+ pre	t_sim	+ pre	t_sim	+ pre	t_sim	+ pre	t_sim	+ pre	t_sim	+ pre
BAR	10	10	10	10	10	10	10	10	10	10	10	10	60	60
TI	10	14	10	14	10	14	10	14	15	19	15	19	70	94
X‐TI	10	10	10	10	10	10	10	10	10	10	10	10	60	60
EDS	3	23	2	22	1	21	> 50	> 64	8	28	> 50	> 90	> 114	> 248
*Multistate methods*														
EDS‐TI	10	14	10	14	3	23	> 50	> 64	10	48	> 61	> 99	> 61	> 99
2D‐TI	100	108	100	108	100	108	100	108	100	108	100	108	100	108
*Self‐convergence*														
EDS	4	24	4	24	1	21	15	29	6	26	9	49	39	173
EDS‐TI	10	14	10	14	2	22	20	34	10	48	41	79	41	79

The “t_sim” column indicates the minimum production run time required to reach convergence, while in the “+ pre” column any necessary preparatory simulation time is added to these values. Coupling parameter methods use 10 *λ*‐points per perturbation.

Overall, for BAR and X‐TI, running 1 ns per *λ*‐value seems to be sufficient to reach convergence. For TI only the KGK‐H2O ↔ KAK‐Dummy and KGK‐Dummy ↔ KAK‐H2O perturbations require elongation at some *λ*‐value due to a large error estimate. This leads to the same conclusion as when investigating the hysteresis (see above) that these are the most challenging perturbations for TI.

The fact that the shape of the TI profile was roughly known beforehand^6^, ^19^ allowed for a favorable spacing of *λ*‐points, giving the coupling parameter‐based methods a certain advantage over EDS. (The spacing was, however, not far from the standard setup of Δ*λ* = 0.1 in most cases (see Table S6 in the Supporting Information). If 2–4 “surplus” *λ*‐points are setup for perturbations that are expected to be difficult (i.e., involving large changes), as we did here, and run at 1 ns each for a first profile estimate, this time might be added to the effective minimum simulation time needed for convergence. We added 4 ns across the board to all TI perturbations in the column “+pre” in Table 3 to account for our pre‐optimization.

For 2D‐TI, the coupling parameter spacing along *λ* remained fixed during the iterative production runs, independent of the value of *κ.* We added 2 x 4 ns in the column “+pre” for 2D‐TI in Table 3 to account for the independent pre‐optimization of *λ* and *κ.*

BAR and X‐TI profit from the same advantage as TI as they were calculated using the same *λ*‐spacing. However, BAR has been shown to be less dependent on the exact placement of the *λ*‐values^28^, ^36^ as long as sufficient overlap remains. In X‐TI the whole profile is predicted from each simulated point; this allows for a rather sparse initial spacing of *λ*‐values (with subsequent placing of alternative or additional values if necessary), and potentially eliminates the need for a preliminary “scan” across *λ* altogether. Thus, we did not add any additional simulation time in the “+pre” column for BAR and X‐TI in Table 3.

EDS needs production run times of just a few nanoseconds for the easier perturbations, but when we take preliminary simulation steps into account, in addition to the pure production run time, the EDS‐based methods emerge as struggling most with our specific test system. EDS‐TI unsurprisingly follows the patterns of each of its parent methods.

Regardless of whether convergence to the BAR reference value was taken or if the self‐convergence was considered, it is clear that for the four easier cases in EDS the vast majority of necessary simulation time was spent on the parameter search. Arguably, suitable EDS parameters could have been found faster if we had chosen a higher initial guess for the *s* parameter, like 0.05. From Figure 3 and Supporting Information Figure S1 we can estimate the time the automated update scheme took to bring *s* from 0.002 to a value of 0.05 which amounts to about 7–8 ns for the nondiagonal perturbations. Possibly the “t_sim” and “+pre” estimates in Table 3 could be reduced by this amount, bringing the total required simulation time down to values that are comparable to TI, but not yet competitive with BAR or X‐TI.

**Figure 6:**
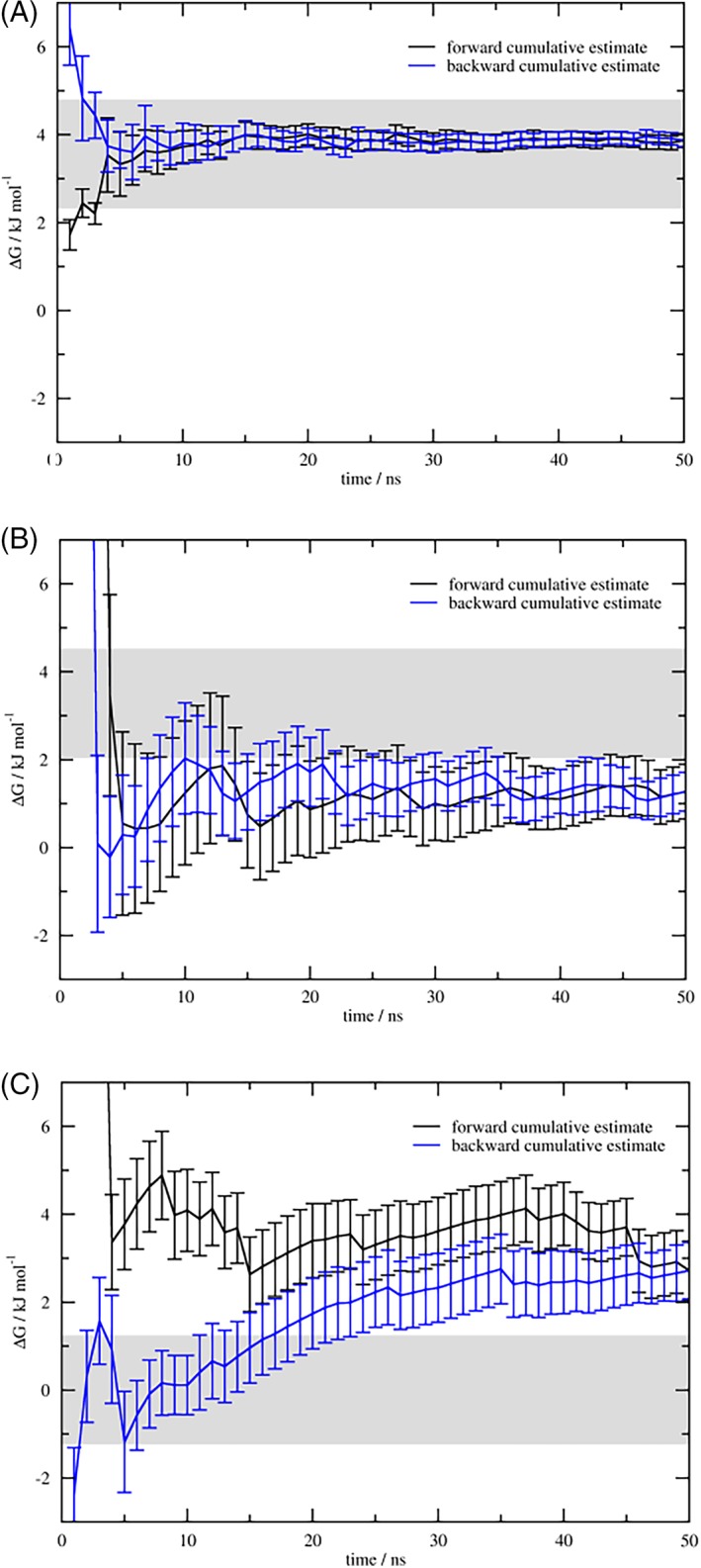
EDS convergence. Cumulative free‐energy estimate for a) KGK‐Dummy ↔ KAK‐Dummy, b) KAK‐H2O ↔ KAK‐Dummy, c) KGK‐Dummy ↔ KAK‐H2O, calculated beginning from the start (black) or the end (blue) of the simulation trajectory. Gray background areas delineate the respective BAR reference value ±½ *k*_B_ *T.* [Color figure can be viewed at http://wileyonlinelibrary.com]

Figure 6 shows the convergence of EDS production simulations in more detail for selected examples. For the remaining perturbations see Figures S2 and S3 in the Supporting Information. The KGK‐Dummy ↔ KAK‐Dummy (GD‐AD) perturbation exemplifies a well‐converging case, as can be seen in panel 6a. Two more difficult cases for EDS are KAK‐H2O ↔ KAK‐Dummy (AH‐AD) and KGK‐Dummy ↔ KAK‐H2O (GD‐AH). Both perturbations do not reach the BAR value within available simulation time, since both seem to be ill‐suited for EDS due to the same steric clash. At times when the system resides in a dummy state, transitions into the real state are energetically unfavorable due to the high repulsive forces caused by overlap of atomic radii. This problem can be observed in both the preliminary parameter search and the production runs for KAK‐H2O ↔ KAK‐Dummy, and is aggravated in the “worst case scenario” KGK‐Dummy ↔ KAK‐H2O.

For KAK‐H2O ↔ KAK‐Dummy, the *s* parameter seemed converged after ~14 ns of parameter search already, but the relatively large fluctuations of the energy offset parameter *E* (see Fig. S1 in the Supporting Information) carry over to the production run as a shift in free energy that cannot be rescued even within the 50 ns of total available simulation time, as can be seen in Figure 6b. When we assess self‐convergence instead of convergence to the final BAR free‐energy value to account for this shift (see last rows in Table 3), the problem does lessen considerably. However, this perturbation still remains the slowest of the set to converge when counting only production run time.

The KAK‐H2O ↔ KAK‐Dummy perturbation using EDS has been repeated in EDS‐TI (albeit with an increased energy offset parameter), as was the KGK‐H2O ↔ KGK‐Dummy perturbation. Convergence behavior, energy distributions, and conclusions are highly similar in each case (see Figs. S2 and S4 in the Supporting Information). In EDS‐TI, the diagonal perturbations are calculated using the respective parts of these two EDS perturbations and a TI perturbation connecting them [see eqs. (19) and (20)]. All of these partial perturbations seem to converge relatively quickly (leading to a KGK‐H2O ↔ KAK‐Dummy perturbation that can compete with TI in terms of production time), except for the KAK‐H2O ↔ KAK‐Reference state partial perturbation. Basically all additional simulation time was spent on this contribution by our prolongation algorithm. The BAR reference value could not be reached within the available simulation time for KGK‐Dummy ↔ KAK‐H2O, and even self‐convergence was the slowest for this perturbation among all investigated methods. Additionally, the preparatory search time needed to find not one but two pairs of EDS parameters increases the total amount of simulation time for the EDS‐TI diagonals massively. The main advantage of EDS‐TI only comes into play when comparing the overall time needed to get free‐energy estimates for all six perturbations at once.

For KGK‐Dummy ↔ KAK‐H2O using EDS, convergence is hindered foremost by the very low *s* parameter. The simulated reference state loses overlap with its constituent end states, and thus, we do not sufficiently sample the end states (see Fig. 5c). When we again assess self‐convergence instead of convergence to the final BAR free‐energy value the problem is lessened as well. While this perturbation remains the slowest of the set to converge overall, the self‐convergence of the production run seems to be comparable to the easier perturbations. However, when examining Figure 6c it becomes clear that while the free‐energy estimate already reached its final value within ½ *k*_B_ *T* after 4 ns, it deviates from it again until 9 ns; only then does it actually converge. The distance restraint setup that is (like for KAK‐H2O ↔ KAK‐Dummy) the cause for the convergence difficulties of the parameter search also leads to a strongly fluctuating free‐energy estimate during the production runs.

Note that while the current setup might seem unphysical and artificial, the underlying problem is not uncommon at all. When the system moves into the KAK‐H2O state, a conformational change needs to take place to release the steric strain. In general, it is quite likely that the conformational preferences for two end states change in a perturbation, and it can be expected that EDS will have difficulty in such cases.

### Predictive analysis: Results at 20 ns total length

Here, we investigate the results of the different free‐energy methods when we prolong simulations solely according to the magnitude of the error estimate. For each of the employed methods we compare in Table 4 how close to the reference value one gets if a maximum of 20 ns simulation time is allowed. As 2D‐TI was only run for 1 ns as a proof‐of‐principle, it is not listed in Table 4.

**Table 4:** Free‐energy differences (kJ mol^‐1^) at 20 ns total simulation length for the individual perturbations (for multistate methods, see text).

	GH‐AH	GD‐AD	GH‐GD	AH‐AD	GH‐AD	GD‐AH
*Pairwise methods*						
BAR	16.4 ± 0.2	3.9 ± 0.3	16.8 ± 0.1	3.2 ± 0.1	20.4 ± 0.3	−0.8 ± 0.1
TI	16.0 ± 0.8	4.0 ± 0.6	16.5 ± 0.6	1.7 ± 0.9	20.2 ± 0.6	0.0 ± 0.9
X‐TI	16.6 ± 0.1	3.7 ± 0.1	17.2 ± 0.2	3.6 ± 0.3	20.2 ± 0.2	−0.8 ± 0.3
EDS^[a]^	17.8 ± 0.6	3.6 ± 0.5	16.8 ± 0.2	0.4 ± 1.7	22.6 ± 0.9	4.6 ± 1.0
*Multistate methods*						
EDS‐TI^[b]^	–	–	–	–	–	–

Coupling parameter methods use 10 *λ*‐points.

[a] Assuming 13 ns of preparatory time and 7 ns of production simulation.

[b] No estimates possible since the combined simulation time always exceeds 20 ns.

In pairwise EDS, the parameter search time comprises the bulk of the total simulation time. As mentioned in the first part of the previous section, this means, at best, a few nanoseconds of production run time. The results given in Table 4 are for 7 ns, which seems quite generous considering that in most cases the parameter search required more than 13 ns. EDS‐TI requires the optimization of two sets of EDS parameters before any production run can take place; this easily uses up more than 20 ns. Thus, no results can be listed for EDS‐TI in Table 4, even if this parameter search time is “shared” among all perturbations.

For the coupling parameter‐based methods, the distribution of available calculation time among the *λ*‐points can be found in Table S7 the Supporting Information. Our simple prolongation algorithm yielded results that were roughly comparable to more elaborate methods.^37^

Within just 20 ns, all remaining methods achieve a result within ½ *k*_B_ *T* of their own final free‐energy estimate for all perturbations, except for EDS on the most difficult test case KGK‐H2O ↔ KAK‐Dummy (GH‐AD). When compared to the BAR reference values, there are more cases that show a larger deviation: One employing TI and three employing EDS. The KAK‐H2O ↔ KAK‐Dummy (AH‐AD) perturbation suffers from the steric clash introduced due to the distance restraint, and both TI and EDS produce a result that is beyond ½ *k*_B_ *T* of this perturbation's BAR reference value. In the most strained test case, KGK‐Dummy ↔ KAK‐H2O (GD‐AH), EDS seems to be a particularly unsuitable method as it displays the largest deviation among all cases, about 2 *k*_B_ *T* off.

The cycle closure for the above simulations is summarized in Table 5. TI performs surprisingly weakly within the limited simulation time of 20 ns, close to the results of the struggling EDS method. One thermodynamic cycle of TI and two cycles of EDS do not close even within 1 *k*_B_ *T.* For EDS, this can be attributed to its difficulty of handling the distance restraint. For TI, it is likely a matter of systematic integration errors. As X‐TI alleviates these integration errors, it performs best in this analysis.

**Table 5:** Free‐energy differences (kJ mol^‐1^) at 20 ns simulation length along the thermodynamic cycles.

	4‐circle	GH‐GD‐AH	GH‐GD‐AD	AH‐AD‐GD	AH‐AD‐GH	Σ	Ω
*Pairwise methods*							
BAR	−1.1 ± 0.4	−0.4 ± 0.3	0.3 ± 0.4	−1.5 ± 0.3	−0.8 ± 0.4	4.1 ± 0.8	0.3 ± 0.2
TI	−2.8 ± 1.4	0.5 ± 1.3	0.4 ± 1.1	−2.3 ± 1.4	−2.4 ± 1.3	8.3 ± 2.9	0.5 ± 0.7
X‐TI	−0.7 ± 0.3	−0.2 ± 0.3	0.6 ± 0.2	−0.9 ± 0.4	−0.1 ± 0.3	2.6 ± 0.7	0.2 ± 0.2
EDS	−2.2 ± 1.9	3.6 ± 1.2	−2.2 ± 1.0	1.4 ± 2.0	−4.4 ± 2.0	13.8 ± 3.8	0.9 ± 0.9

Simulation data is taken from Table 4. Coupling parameter methods use 10 *λ*‐points.

The sum of all deviations from cycle closure *Σ* as well as the average deviation per perturbation Ω are smallest (best) for X‐TI and BAR and largest (worst) for EDS. BAR shows slightly larger deviations than X‐TI within the limited simulation time. All individual thermodynamic cycles of BAR and X‐TI close within ½ *k*_B_ *T*, except the KAK‐H2O ↔ KAK‐Dummy ↔ KGK‐Dummy (AH‐AD‐GD) case using BAR (a cycle containing both of the most difficult perturbations), which closes just slightly beyond ½ *k*_B_ *T.*

### Practical considerations and usability

Despite all theoretical elegance, the practical implementation and usability of a method will decide to a good deal how widely spread it is in its use. Similarly, convergence speed considerations must not just take pure calculation time into account, but also the number of preparatory steps or parameters to be optimized. These factors differ between the investigated methods.

All coupling parameter‐based methods require a decision on the user's side about which values of *λ* to simulate at. An advantage of BAR is that it is rather robust concerning this *λ*‐spacing.^28^, ^36^ Another advantage is that some programs (e.g., when optimized for speed) do not supply calculation of free‐energy derivatives as would be needed in TI and X‐TI; for these BAR represents a relatively easy postprocessing step.^38^ However, a reevaluation of the energies is required if alternative *λ*‐values are found necessary later on, leading to extensive postprocessing of trajectories if these energies are not provided on the fly. For this reason, it remains a relevant question if alternative more user‐friendly methods are of comparable efficiency in practice.

The efficiency and accuracy of the TI method depends on how exactly the end states are connected. Too widely spaced *λ*‐points cause insufficient overlap between the sampled conformational spaces and a systematic error on integration. Too small spacing wastes computational resources. This dependence on a user specification of *λ* necessitates a certain amount of preparatory effort, for example, a short sweep over a generous amount of *λ*‐points for a first estimate of the TI profile. Additionally, dedicated code to calculate the derivatives of the free energy is necessary to run TI. However, as of the time of writing, most major MD programs supply such code^38^; thus, TI (together with BAR) remains one of the most widely used free‐energy methods.

**Figure 7:**
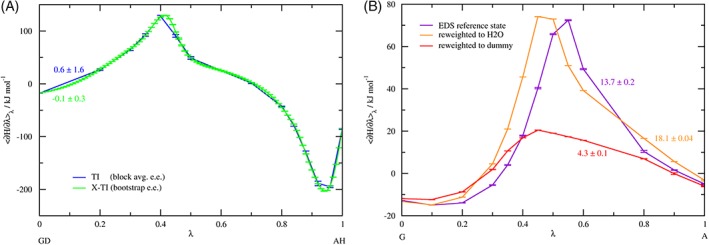
Characteristic TI profiles, using the full number of *λ*‐points. a) Comparison of the TI and X‐TI profile for KGK‐Dummy ↔ KAK‐H2O (GD‐AH), at 1 ns simulation time per *λ*‐point. b) EDS‐TI profiles obtained (after 50 ns simulation time per *λ*‐point) by reweighting the EDS reference state that was propagated along *λ* to the respective water end states. The numbers next to the lines give the integrated free‐energy difference in kJ mol^−1^. [Color figure can be viewed at http://wileyonlinelibrary.com]

As X‐TI predicts the values of 〈∂H/∂*λ*〉_*λ*_ over an arbitrarily large number of *λ*‐points (101 in our case), the resulting free‐energy profile is smoother than a regular TI profile, which usually sports only about 1/10th this amount of *λ*‐points. Figure 7 shows for the KGK‐Dummy ↔ KAK‐H2O (GD‐AH) perturbation that TI indeed suffers from integration errors when compared to the X‐TI profile. These errors are somewhat reduced due to a fortuitous cancellation of errors, but will remain even in the limit of infinite sampling.

The overhead for predicting the values of 〈∂H/∂*λ*〉_*λ*_ during all simulations for all other *λ*‐points is small in in terms of computation time, and creates a negligible amount of additional output data.^10^ Compared to the resources needed to actually simulate at additional *λ*‐points, this method provides a definite practical advantage over TI. Furthermore, a first sweep over *λ* becomes obsolete as a reasonably accurate curve can be estimated from only a few *λ*‐points, directly suggesting which *λ*‐points to add.

Because in our current implementation of X‐TI all necessary energies and energy‐derivatives are computed on the fly, adding new *λ*‐values to a preliminary set of simulations can be done without significant re‐evaluation of existing trajectories, as may be necessary for BAR.

**Figure 8:**
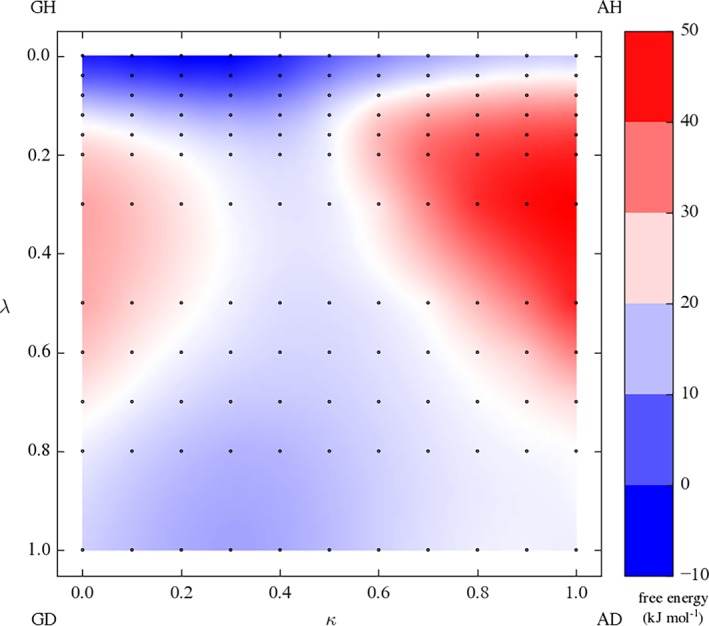
Free‐energy surface of 2D‐TI. Each dot represents one production simulation. [Color figure can be viewed at http://wileyonlinelibrary.com]

The 2D‐TI approach offers the complete free‐energy landscape between the four end states, which comes at the cost of simulating a very large number of unphysical intermediates. It is, therefore, not expected to be competitive in terms of efficiency. A theoretical advantage is that it allows a user to identify more optimal paths connecting the end states along which the simulation could be setup. From Figure 8 a narrow path on the free‐energy landscape could be identified for the KGK‐Dummy ↔ KAK‐H2O (GD‐AH) transition that involves very little changes in the free‐energy derivatives.

The main advantage of EDS lies in the generation of an optimal reference state Hamiltonian. This avoids the dependence of the coupling parameter methods on user specification. As sampling is focused on the important parts of phase space and unnecessary intermediate states are avoided, the EDS method would realize its full potential not in a pairwise setup of states but with multiple states included into the reference state simulation (see the section on Multistate EDS).

The main drawback of EDS is that the computational effort shifts to the preparatory phase, the optimization of the *E* and *s* parameters necessary for the construction of an efficient reference state. This optimization and reference state construction presents a somewhat increased level of complexity to the user compared to the coupling parameter methods; as such we label the EDS method a theoretically elegant tool for the advanced user.

In addition, as an EDS simulation in principle switches between end states serially, the simulation time required increases linearly with the number of states. This is necessary to gather meaningful statistics and sample all states equally. As a consequence, the fact that EDS condenses a multidimensional problem into a single simulation means it is no longer as easily parallelizable as the coupling parameter methods, which basically run a completely independent simulation at each employed value of *λ.* Thus, the local computer architecture of the user becomes an additional deciding factor: A single powerful GPU in principle lends itself better to EDS calculations than a collection of weaker CPUs.

As EDS‐TI is a hybrid method, it inherits the benefits and drawbacks of each of its parent methods. Substantial advantages of EDS‐TI are that (a) it can be parallelized like TI, and (b) once setup, one single implementation can provide several free‐energy differences at once. We initially expected that the EDS‐TI approach would be a particularly elegant way of handling this system, but conclude that this is not the case. The main disadvantage is that the EDS part needs careful preliminary parameter optimization and has difficulties handling a conformational difference between the end states. Furthermore, as in TI and especially 2D‐TI, the bulk of computation time is spent on (unphysical) intermediate and reference states, reducing the physical relevance of the simulations. Moreover, the TI profile needs to be reweigthed to correspond to the EDS end states, adding noise to the calculation.

The final TI profiles obtained from EDS‐TI for the water/dummy end states can be found in Figure 7b above. Future applications of this method might be efficient if the system can be setup such that EDS does not need to handle a conformational change.

### Multistate EDS

This work is not the first to address the problem of alchemical changes that appear concurrent with a change in the hydration state of an active site.^39^ The EDS‐TI approach is somewhat reminiscent of the grand‐canonical Monte Carlo approach described by Ross et al.^40^ In this approach, water molecules are added or removed during an alchemical modification.

A further possible calculation method that has not been addressed in the current work yet is multistate EDS (mEDS), which is a generalization of EDS for more than two end states.^17^) In pairwise methods $\left(\begin{array}{c} N_{H}\\ 2 \end{array}\right)=\frac{1}{2}N_{H}\left(N_{H}-1\right)$ free‐energy simulations are performed. In contrast, an mEDS calculation relies on a single simulation during which the constituting Hamiltonians *H*_*i*_ in eq. (16) are computed. As the *H*_*i*_s typically differ only in a limited number of interactions, an mEDS calculation would in principle always be more efficient than many individual simulations in which all interactions are to be computed. In other words, the EDS methodology is expected to be increasingly more efficient than the other methods with an increasing number of end states *N*_*H*_.

The main advantage of mEDS would, thus, be that it conveniently gathers conformations relevant to multiple end states during just a single simulation. This means a gain in efficiency on the user's side if larger numbers of end states are to be investigated, which is not the case in the present work. However, the quest for appropriate parameters would get more cumbersome with each end state added.

Several possibilities exist to connect multiple end‐state Hamiltonians into an mEDS reference state.^31^ Due to its all‐in‐one character, mEDS needs even more carefully optimized parameters than EDS to ensure balanced sampling. An efficient parameter search scheme is required as the effects of the energy offsets and smoothness parameter on the energy surface of the reference state are coupled.

Instead of searching for optimal parameters, the replica exchange (RE) methodology^41^, ^42^, ^43^ can be used to sample at a whole range of different *s* values in parallel.^34^, ^44^ Preliminary simulations suggested that no single set of *s* and *E*_*i*_ values could be found for an efficient mEDS simulation and that a RE setup would be necessary with the current system too (data not shown). However, this comes at additional computational costs, also in the production run. This means that as long as the number of necessary replicas is larger than the number of investigated end states, RE‐mEDS cannot perform more efficiently than the pairwise methods. Also, in terms of applicability, any advantages or disadvantages of the EDS method will be inherited by mEDS as well. We have, therefore, not explored this possibility further in this work.

## Conclusion

Here, we have used over 4 μs of total simulation time to investigate the ability of several free‐energy methods to handle a simple model system mimicking the removal of water molecules during an alchemical change. In the perturbations KGK‐H2O ↔ KAK‐H2O, KGK‐Dummy ↔ KAK‐H2O, KAK‐H2O ↔ KAK‐Dummy, this model system shows a steric clash, which results in extending the distance between C_β_ and the water molecule slightly (against the distance restraint). This makes the model system more generally representative of alchemical free‐energy calculations that are leading to conformational changes along the way.

The steric clash introduced into the system by distance restraining a water molecule to the exact position of alanine's beta carbon lead to grave difficulties in finding useful EDS parameters. Due to the hybrid nature of the simulated reference state, EDS seems to be the least suitable method for the investigated system. More generally, it appears to be a problem of EDS that any system that undergoes large conformational changes from one end state to another (such as helix inversion,^45^ helix type change,^46^ or a domain's hinge motion) will be quite challenging. It is, however, noteworthy that our specific setup, intended to mimic the replacement of a binding‐site water by an aliphatic side chain, represents a case with difficulties unseen in some other applications (such as binding of several similar ligands to a common receptor.^33^)

In this respect, a simple TI calculation seemed already more efficient for this model system although it suffers from systematic integration errors, especially for the most difficult perturbations. X‐TI alleviates these errors, and seems to be as quickly converging as the BAR calculations. While BAR remains the theoretically most efficient method, X‐TI comes with the additional practical advantage that a quick estimate of the entire profile is already available after just a few simulations, from which an educated guess can be made as to which *λ*‐values to add to the set. Of course, it cannot be excluded that for different systems with other objectives, different methods show increased efficiency.

Returning to the original aims of this work, we investigated if alchemical calculations of binding free energies could be combined with estimates on the presence or absence of water molecules in the active site. From a careful analysis of the model system described here, we conclude that setups in which multiple free energies are computed simultaneously (2D‐TI, EDS‐TI) are not competitive compared to simply performing the necessary pairwise free‐energy calculations using, for example, BAR or X‐TI.

## Author contributions

The manuscript was written by MM. All simulations were conducted by MM. NH supervised the EDS simulations and manuscript writing. CO supervised all other simulations and manuscript writing.

## Supporting information


**Table S1:** Data storage settings
**Table S2:** EDS parameters
**Table S3:** Maximum production run times (ns) for the individual perturbations
**Table S4:** Maximum production run times (ns) along the thermodynamic cycles
**Table S5:** TI hysteresis (kJ mol^−1^)
**Table S6:** λ‐points overview
**Table S7:** Distribution of 20 ns available simulation time among λ‐points
**Figure S1:** EDS parameter evolutions for all perturbations during the prolonged automated search procedure.
**Figure S2:** EDS‐TI convergence, EDS part
**Figure S3:** EDS convergence
**Figure S4:** EDS energy distributions
**Figure S5:** TI profile for KGK‐H2O ↔ KAK‐Dummy at 1 ns per λ‐point.
