# Supplementary material for: Comparison of free‐energy methods using a tripeptide‐water model system
Source: J Comput Chem. 2018 Oct 2;39(26):2226–42. doi: 10.1002/jcc.25537 (PMC6220940; doi:10.1002/jcc.25537)
Supplement: Supplementary file 1 — Table S1: Data storage settings Table S2: EDS parameters Table S3: Maximum production run times (ns) for the individual perturbations Table S4: Maximum production run times (ns) along the thermodynamic cycles Table S5: TI hysteresis (kJ mol−1) Table S6: λ‐points overview Table S7: Distribution of 20 ns available simulation time among λ‐points Figure S1: EDS parameter evolutions for all perturbations during the prolonged automated search procedure. Figure S2: EDS‐TI convergence, EDS part Figure S3: EDS convergence Figure S4: EDS energy distributions Figure S5: TI profile for KGK‐H2O ↔ KAK‐Dummy at 1 ns per λ‐point. [file JCC-39-2226-s001.pdf]

Supporting Information

for

Comparison of Free-Energy Methods Using a Tripeptide-Water Model System

Manuela Maurer[1], Niels Hansen[2], and Chris Oostenbrink[1*]

[1] Institute of Molecular Modeling and Simulation, University of Natural Resources and Life Sciences, Muthgasse 18, A-1190 Vienna, Austria
[2] Institute of Thermodynamics and Thermal Process Engineering, Pfaffenwaldring 9, 70569 Stuttgart, Germany
* Corresponding author. E-mail: chris.oostenbrink@boku.ac.at

Contents:

Table S1: Data storage settings

Table S2: EDS parameters

Table S3: Maximum production run times (ns) for the individual perturbations

Table S4: Maximum production run times (ns) along the thermodynamic cycles

Table S5: TI hysteresis (kJ mol^{-1})

Table S6: λ-points overview

Table S7: Distribution of 20 ns available simulation time among λ-points

Figure S1: EDS parameter evolutions for all perturbations during the prolonged automated search procedure.

Figure S2: EDS-TI convergence, EDS part

Figure S3: EDS convergence

Figure S4: EDS energy distributions

Figure S5: TI profile for KGK-H2O ↔ KAK-Dummy at 1 ns per λ-point.

Tables

SI_Table 1: *Data storage settings. Coordinates, energies and λ-derivatives of the energies were written to disc every indicated number of steps.*

method	coordinates	energies	free energies
BAR, X-TI	5000 steps (10 ps)	20 steps (0.04 ps)	20 steps (0.04 ps)
TI	-	500 steps (1 ps)	500 steps (1 ps)
2D-TI	100 steps (0.2 ps)	50 steps (0.01 ps)	50 steps (0.01 ps)
EDS	-	100 steps (0.02 ps)	-
EDS-TI	1000 steps (2 ps)	100 steps (0.02 ps)	100 steps (0.02 ps)

SI_Table 2: *EDS parameters. Values found by the automated parameter search scheme, and runtimes of each search.*

	GH - AH	GD – AD	GH - GD	AH - AD	GH - AD	GD - AH
time (ns)						
maximum search length	40	100	40	40	100	100
production started after	20	20	20	20	20	40
plateau for *s* reached after	19.4	> 100	19.6	13.8	50.4	> 100
value of s						
value used for production	0.330	0.532	0.077	0.058	0.049	0.035
value at plateau	0.330	0.782	0.077	0.058	0.085	0.03 to 0.055
value of E *(kJ mol^{-1})*						
value used for production	13.9	4.6	24.5	8.5	23.8	-44.5
value at plateau	15.4	~ 4	24.1	~ 14.5	21.2	-

SI_Table 3: *Maximum production run times (ns) for the individual perturbations*

		GH - AH		GD - AD		GH - GD		AH - AD		GH - AD		GD - AH		all 6	
pairwise methods															
	ns / λ	λ-points	t_sim	λ-points	t_sim	λ-points	t_sim	λ-points	t_sim	λ-points	t_sim	λ-points	t_sim	λ-points	t_sim
**BAR**	20	12	240	10	200	14	280	14	280	14	280	14	280	78	1560
**TI**	20	12	240	10	200	14	280	14	280	14	280	14	280	78	1560
**X-TI**	20	12	240	10	200	14	280	14	280	14	280	14	280	78	1560
**EDS**	-	-	50	-	50	-	50	-	50	-	50	-	50	-	300
multistate methods															
**EDS-TI**	50	13	650	13	650	-	50	-	50	13	650	13	650	13	650
**2D-TI**	1	11	11	11	11	12	12	12	12	132	12	132	12	132	132

SI_Table 4: *Maximum production run times (ns) along the thermodynamic cycles*

		4-circle		GH-GD-AH		GH-GD-AD		AH-AD-GD		AH-AD-GH		Σ		Ω	
pairwise methods															
	ns / λ	λ-points	t_sim	λ-points	t_sim	λ-points	t_sim	λ-points	t_sim	λ-points	t_sim	λ-points	t_sim	λ-points	t_sim
**BAR**	20	50	1000	40	800	38	760	38	760	40	800	206	1560	13	258
**TI**	20	50	1000	40	800	38	760	38	760	40	800	206	1560	13	258
**X-TI**	20	50	1000	40	800	38	760	38	760	40	800	206	1560	13	258
**EDS**	-	-	200	-	150	-	150	-	150	-	150	-	300	-	50
multistate methods															
**EDS-TI**	50	13	650	13	650	13	650	13	650	13	650	13	650	4	206
**2D-TI**	1	46	46	35	35	35	35	35	35	35	35	70	70	12	12

SI_Table 5: *TI hysteresis (kJ mol^{-1}). Quality assessment by reversing a TI perturbation just conducted and comparing the independent forward and backward free-energy estimates (kJ mol^{-1}). The results are given for minimum (10 λ-points with 1 ns each) and maximum (see Table S3) simulation lengths.*

	GH - AH			GD - AD			GH - GD			AH - AD			GH - AD			GD - AH		
maximum simulation length																		
**forward**	16.9	±	0.4	3.6	±	0.3	16.5	±	0.3	2.3	±	0.3	20.3	±	0.4	1.2	±	0.4
**backward**	-17.2	±	0.4	-3.5	±	0.3	-16.5	±	0.3	-2.1	±	0.3	-20.6	±	0.5	-1.6	±	0.6
**hysteresis**	0.3			0.1			0.1			0.1			0.3			0.4		
minimum simulation length																		
**forward**	16.3	±	1.2	4.0	±	0.8	16.5	±	0.9	2.2	±	1.2	19.8	±	1.3	-0.2	±	1.6
**backward**	-16.8	±	1.0	-4.5	±	1.1	-15.3	±	0.8	-2.1	±	1.3	-21.7	±	1.6	0.9	±	1.2
**hysteresis**	0.4			0.5			1.2			0.02			1.9			0.7		

SI_Table 6: *λ-points overview. Values of the coupling parameter λ used in simulations and analysis for each of the six perturbations.*

**KGK-H2O ↔ KAK-H2O**	λ-points													
method	0.000	0.100	0.200	0.300	0.350	0.400	0.450	0.500	0.550	0.600	0.700	0.800	0.900	1.000
BAR, X-TI	x	x	x	x		(x)	x	(x)	x	x	x	x		x
TI	x	x	x	x		(x)	x	(x)	x	x	x	x		x
2D-TI	x	x	x	x		x		x		x	x	x	x	x
EDS-TI	x	(x)	x	x	x	(x)	x	x	x	x		x	(x)	x

**KGK-Dum ↔ KAK-Dum**	λ-points													
method	0.000	0.100	0.200	0.300	0.350	0.400	0.450	0.500	0.550	0.600	0.700	0.800	0.900	1.000
BAR, X-TI	x	x	x	x		x		x		x	x	x		x
TI	x	x	x	x		x		x		x	x	x		x
2D-TI	x	x	x	x		x		x		x	x	x	x	x
EDS-TI	x	(x)	x	x	x	(x)	x	x	x	x		x	(x)	x

**KGK-H2O ↔ KGK-Dum**	λ-points													
method	0.000	0.040	0.080	0.120	0.160	0.200	0.300	0.500	0.600	0.650	0.700	0.750	0.800	1.000
BAR, X-TI	x	x	x	(x)	x	x	x	(x)	x	(x)	x	x	(x)	x
TI	x	x	x	(x)	x	x	x	(x)	x	(x)	x	x	(x)	x
2D-TI	x	x	x	x	x	x	x	x	x		x		x	x
EDS-TI	not applicable for EDS													

**KAK-H2O ↔ KAK-Dum**	λ-points													
method	0.000	0.040	0.080	0.120	0.160	0.200	0.300	0.500	0.600	0.650	0.700	0.750	0.800	1.000
BAR, X-TI	x	x	x	(x)	(x)	x	x	(x)	x	x	x	(x)	x	x
TI	x	x	x	(x)	(x)	x	x	(x)	x	x	x	(x)	x	x
2D-TI	x	x	x	x	x	x	x	x	x		x		x	x
EDS-TI	not applicable for EDS													

**KGK-H2O ↔ KAK-Dum**	λ-points													
method	0.000	0.040	0.080	0.120	0.160	0.200	0.300	0.500	0.600	0.650	0.700	0.750	0.800	1.000
BAR, X-TI	x	x	x	(x)	(x)	x	x	x	x	x	(x)	(x)	x	x
TI	x	x	x	(x)	(x)	x	x	x	x	x	(x)	(x)	x	x
2D-TI	all of KGK-H2O ↔ KAK-H2O and KAK-H2O ↔ KAK-Dum													
EDS-TI	see KGK-H2O ↔ KAK-H2O													

**KGK-Dum ↔ KAK-H2O**	λ-points													
method	0.000	0.200	0.300	0.350	0.400	0.450	0.500	0.700	0.800	0.840	0.880	0.920	0.960	1.000
BAR, X-TI	x	x	x	(x)	x	(x)	x	x	x	(x)	(x)	x	x	x
TI	x	x	x	(x)	x	(x)	x	x	x	(x)	(x)	x	x	x
2D-TI	all of KGK-Dum ↔ KAK-Dum and KAK-H2O ↔ KAK-Dum													
EDS-TI	see KGK-Dum ↔ KAK-Dum													

x = simulated, used in analysis
(x) = simulated, removed for analysis

SI_Table 7: *Distribution of 20 ns available simulation time among λ-points. Methods not mentioned here either produce only a single trajectory or could not provide a result within 20 ns total time.*

KGK-H2O ↔ KAK-H2O

method	λ-points									
	0.000	0.100	0.200	0.300	0.450	0.550	0.600	0.700	0.800	1.000
BAR	1	3	3	1	2	3	3	2	1	1
X-TI	1	1	1	1	5	4	2	2	2	1
TI	1	1	1	3	8	2	1	1	1	1

KGK-Dum ↔ KAK-Dum

method	λ-points									
	0.000	0.100	0.200	0.300	0.400	0.500	0.600	0.700	0.800	1.000
BAR	1	1	2	5	4	2	2	1	1	1
X-TI	1	1	1	1	3	4	3	2	3	1
TI	1	1	1	1	1	8	4	1	1	1

KGK-H2O ↔ KGK-Dum

method	λ-points									
	0.000	0.040	0.080	0.160	0.200	0.300	0.600	0.700	0.750	1.000
BAR	2	3	3	2	1	1	2	2	2	2
X-TI	1	1	3	1	1	3	5	1	3	1
TI	1	1	3	1	1	1	6	1	4	1

KAK-H2O ↔ KAK-Dum

method	λ-points									
	0.000	0.040	0.080	0.200	0.300	0.600	0.650	0.700	0.800	1.000
BAR	1	2	4	3	1	2	3	2	1	1
X-TI	1	1	3	2	2	6	2	1	1	1
TI	1	1	3	2	1	6	2	1	2	1

KGK-H2O ↔ KAK-Dum

method	λ-points									
	0.000	0.040	0.080	0.200	0.300	0.500	0.600	0.650	0.800	1.000
BAR	1	1	1	1	6	6	1	1	1	1
X-TI	1	1	5	2	2	4	1	1	2	1
TI	1	2	3	3	3	2	1	1	3	1

KGK-Dum ↔ KAK-H2O

method	λ-points									
	0.000	0.200	0.300	0.400	0.500	0.700	0.800	0.920	0.960	1.000
BAR	1	2	3	3	2	1	2	3	2	1
X-TI	1	1	1	3	4	2	2	4	1	1
TI	1	2	2	2	4	2	1	4	1	1

Figures

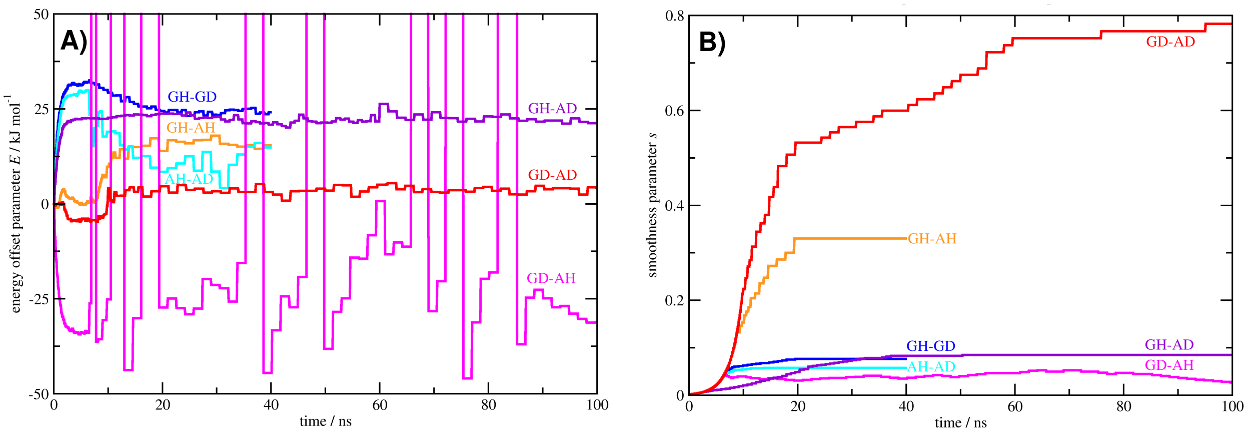

SI_Figure 1: *EDS parameter evolutions for all perturbations during the prolonged automated search procedure. A)* E *parameter, B)* s *parameter.*

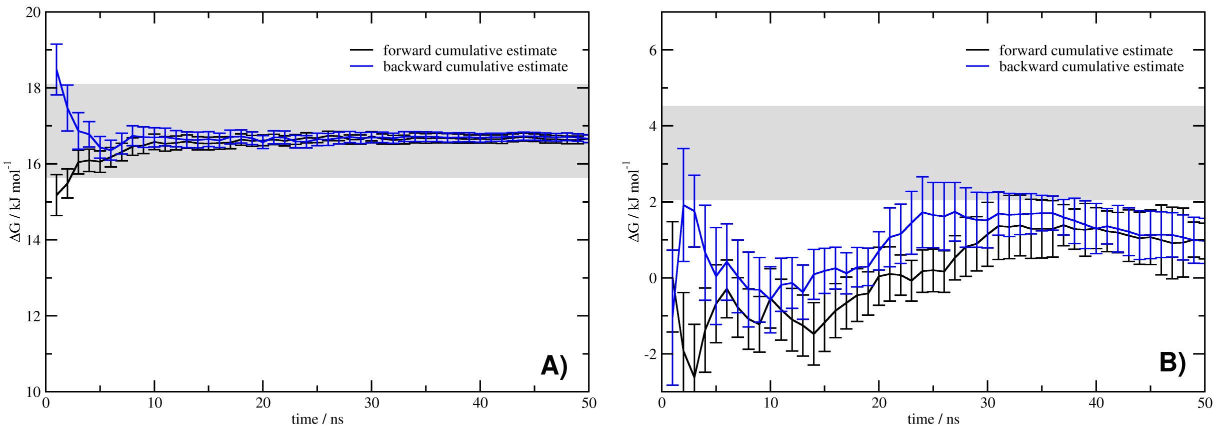

SI_Figure 2: *EDS-TI convergence, EDS part. Cumulative free-energy estimate for A) KGK-H2O ↔ KGK-Dummy, B) KAK-H2O ↔ KAK-Dummy, calculated beginning from the start (black) or the end (blue) of the simulation trajectory. Gray background areas delineate the respective BAR reference value ± ½* k_BT.

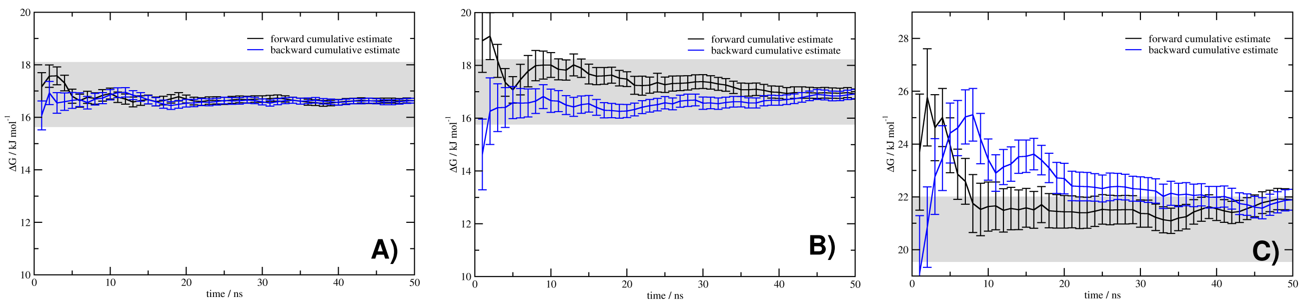

SI_Figure 3: *EDS convergence. Cumulative free-energy estimate for A) KGK-H2O ↔ KGK-Dummy, B) KGK-H2O ↔ KAK-H2O, C) KGK-H2O ↔ KAK-Dummy, calculated beginning from the start (black) or the end (blue) of the simulation trajectory. Gray background areas delineate the respective BAR reference value ±* ½ k_BT.

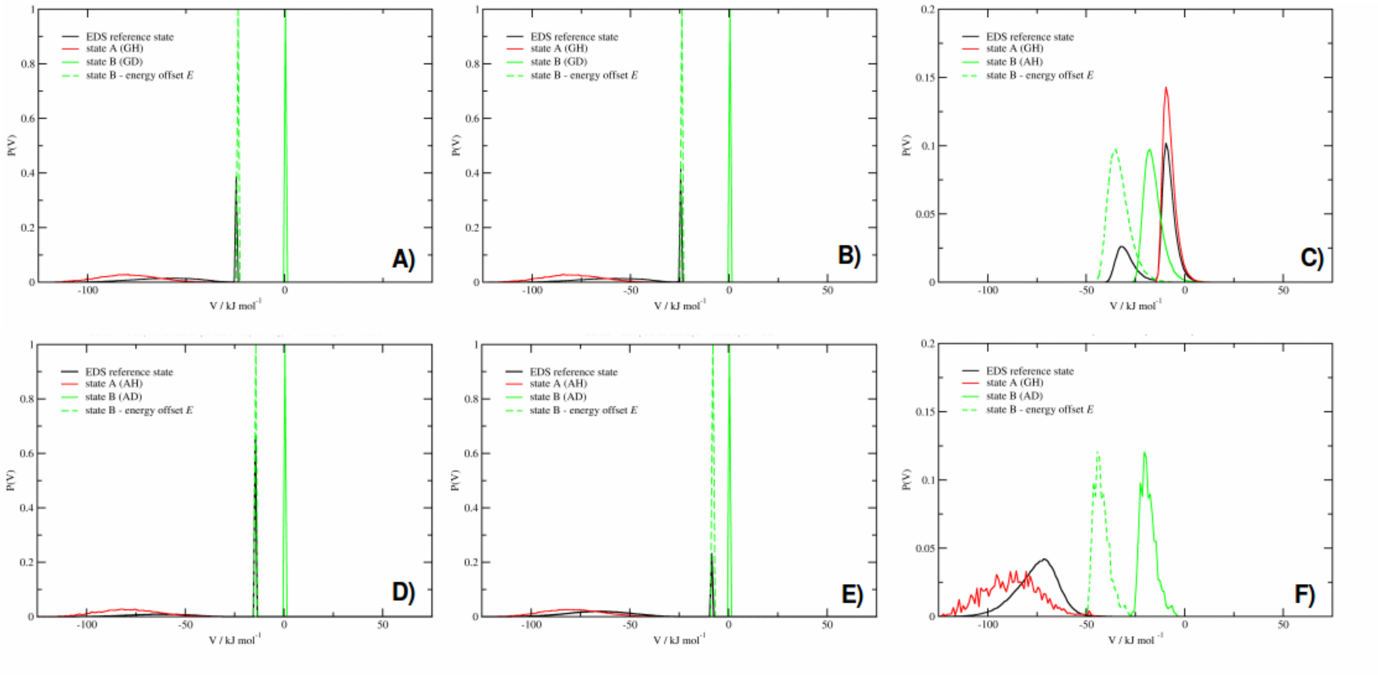

SI_Figure 4: *EDS energy distributions. Potential energy of the perturbed atoms during 50 ns production run for: Left column: the EDS part of EDS-TI. Central and right column: EDS. A) and B): KGK-H2O ↔ KGK-Dummy (GH-GD); D) and E): KAK-H2O ↔ KAK-Dummy (AH-AD). C) KGK-H2O ↔ KAK-H2O (GH-AH). F) KGK-H2O ↔ KAK-Dummy (GH-AD). The distribution of the EDS reference state (black) is compared to the reweighted energy distributions of the end states (red and green).*

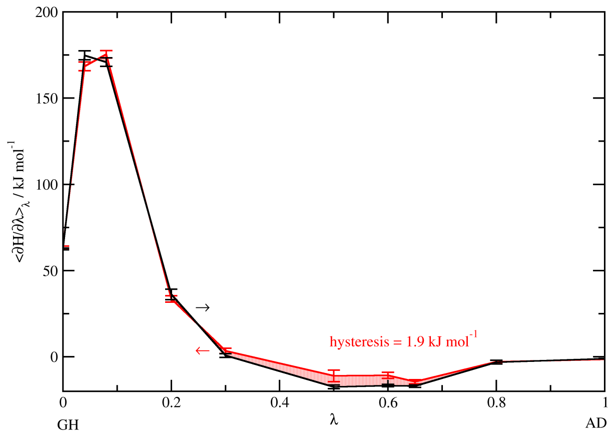

SI_Figure 5: *TI profile for KGK-H2O ↔ KAK-Dummy at 1 ns per λ-point. The red hatched area between forward (black) and backward (red) perturbations represents the hysteresis, which is the largest of the set at this simulation length.*
